# A comprehensive benchmarking of machine learning algorithms and dimensionality reduction methods for drug sensitivity prediction

**DOI:** 10.1093/bib/bbae242

**Published:** 2024-05-26

**Authors:** Lea Eckhart, Kerstin Lenhof, Lisa-Marie Rolli, Hans-Peter Lenhof

**Affiliations:** Center for Bioinformatics, Saarland Informatics Campus, Saarland University, 66123, Saarland, Germany; Center for Bioinformatics, Saarland Informatics Campus, Saarland University, 66123, Saarland, Germany; Center for Bioinformatics, Saarland Informatics Campus, Saarland University, 66123, Saarland, Germany; Center for Bioinformatics, Saarland Informatics Campus, Saarland University, 66123, Saarland, Germany

**Keywords:** drug sensitivity prediction, machine learning, dimension reduction, feature selection, feature extraction, cancer cell lines

## Abstract

A major challenge of precision oncology is the identification and prioritization of suitable treatment options based on molecular biomarkers of the considered tumor. In pursuit of this goal, large cancer cell line panels have successfully been studied to elucidate the relationship between cellular features and treatment response. Due to the high dimensionality of these datasets, machine learning (ML) is commonly used for their analysis. However, choosing a suitable algorithm and set of input features can be challenging. We performed a comprehensive benchmarking of ML methods and dimension reduction (DR) techniques for predicting drug response metrics. Using the Genomics of Drug Sensitivity in Cancer cell line panel, we trained random forests, neural networks, boosting trees and elastic nets for 179 anti-cancer compounds with feature sets derived from nine DR approaches. We compare the results regarding statistical performance, runtime and interpretability. Additionally, we provide strategies for assessing model performance compared with a simple baseline model and measuring the trade-off between models of different complexity. Lastly, we show that complex ML models benefit from using an optimized DR strategy, and that standard models—even when using considerably fewer features—can still be superior in performance.

## Introduction

Cancerous diseases are characterized by geno- and phenotypic heterogeneity impacting the success of chemotherapeutic treatments. Consequently, one major goal of precision oncology is to identify the most efficient drug candidates and to detect biomarkers affecting treatment response.

Cancer cell line panels like the *Genomics of Drug Sensitivity in Cancer* (GDSC) [1] and *Cancer Cell Line Encyclopedia* (CCLE) [2] provide multi-omics measurements and drug response metrics for various cancer types and can be used to study the relationship between cellular features and treatment outcome. Due to the high dimensionality of these datasets, machine learning (ML) is often applied for their analysis [3–34]. This typically involves the prediction of drug response measures and the inference of sensitivity- or resistance-related biomarkers.

Increasingly complex models such as deep neural networks with carefully constructed architectures [4, 19, 24, 26, 30, 32] and advanced tree-based models [8, 16] have shown promising results recently. However, Li *et al*. [35] found that many deep learning approaches are not superior in performance to simple feed-forward neural networks or random forests for drug sensitivity prediction. Apart from that, keeping models as simple and interpretable as possible is desirable for studying the relation between cellular features and drug response and creating trustworthy, comprehensible predictions to enable ML use for clinical decision support. This highlights the need to investigate how complex models must be given the current data situation and how even standard models can be elevated through hyperparameter tuning and choosing informative inputs. Moreover, given the availability of programming packages such as caret [36] and scikit-learn [37], basic ML models can even be trained by non-experts, rendering them a particularly attractive starting point for model development.

When ML is used to investigate high-dimensional datasets such as the GDSC or CCLE data, it is typically indispensable to perform dimension reduction (DR) (i.e. to reduce the number of input features) to counteract the *curse of dimensionality*, reduce runtime and potentially increase model interpretability. Two groups of DR algorithms can be distinguished: feature selection (FS) and feature extraction (FE) techniques [38]. FS algorithms aim to choose a subset of interesting features from a given feature set. They can be grouped into filters, wrappers and embedded methods [38]. Filter methods can be seen as a pre-processing step, where features are chosen before applying any ML algorithm. In contrast, wrappers train ML models using different feature sets and select the best features based on model error. Lastly, embedding methods are ML algorithms like elastic net (cf. Methods) where FS is integrated into the model training. In contrast to FS, FE aims to transform a high-dimensional dataset into a lower dimensional representation, which still retains the properties of the original data space. While FE does not require discarding potentially valuable features, the resulting low-dimensional data representation is often not easily interpretable, as each calculated feature is a combination of features from the original feature space. Both FS and FE are commonly used for drug sensitivity prediction (cf. Supplementary Table 1 which lists the used DR methods used for 32 state-of-the-art methods): one straightforward FS approach is choosing features with strongest correlation to the response [23, 39]. Alternatively, many approaches only consider features of literature-derived oncogenes as predictors [4, 5, 7, 11, 19, 22, 25, 26, 29], while Su *et al*. [8] use randomly selected features.

Regarding FE, principal component analysis (PCA) was employed by several contestants of the DREAM7 drug sensitivity prediction challenge [39], while others utilize autoencoders [3, 25, 31, 33, 34] and Wang *et al*. [10] employ a matrix factorization approach. A biologically more interpretable FE approach is to summarize gene-level data into scores for the corresponding molecular pathways, as done by Tang *et al*. [9] and Karagiannaki *et al*. [40].

Given the plethora of available ML algorithms and DR techniques, it is challenging to select the right combination of methods. Unfortunately, our literature research revealed that publications often do not report whether different approaches were tested or how they performed in comparison (cf. Supplementary Table 1) highlighting the need for a systematic assessment. There exist several insightful reviews and evaluations on ML and DR in drug sensitivity prediction [41–45]: Chen *et al*. [41] benchmarked several state-of-the-art deep learning approaches but did not investigate different DR methods. Jang *et al*. [42] analyzed the performance of different input features and prediction algorithms. However, they focused on inputs from varying omics-types rather than different DR techniques. Koras *et al*. [43] compared several FS methods but did not account for size differences in the investigated feature sets, which might impact performance more significantly than the actual features. Considering the goal of having accurate but interpretable models, it is crucial to investigate whether small feature sets are sufficient to train good predictors and how they compare with larger sets.

In this paper, we discuss how the problem of drug sensitivity prediction can be addressed using four different ML algorithms in combination with nine DR techniques. Using gene expression values and drug response measures from the GDSC, we trained more than 16 000 000 models for 179 compounds and compared the results regarding prediction accuracy, runtime and interpretability.

Our findings reveal that the choice of both the ML algorithm and DR method have substantial impact on prediction performance. For most drugs, elastic net models had the best performance and lowest runtime, while neural networks performed worst. The best-performing DR methods were PCA and a heuristic by Kwak and Choi [46] that is based on the *minimum-redundancy-maximum-relevance* principle. Overall, FS methods considering the drug response performed better than methods using only expression values.

We also discuss how performance trade-offs between models and for different numbers of input features can be assessed. Furthermore, we show how cross-validation (CV) can be used to bias models to improve predictions for the most sensitive cell lines, which are typically highly underrepresented in drug screens [5, 11, 16]. To account for the goal of interpretable models, we characterize the four investigated ML algorithms in terms of model transparency [47] and explainability [48]. We also show how the selected features for each drug can already present valuable insight into drug responses even without applying any ML algorithm. Lastly, our analyses using a multi-omics multi-task deep learning approach by Chiu *et al*. [3] prove that complex prediction models (1) can benefit substantially from using different DR methods and (2) can be outperformed by standard models even with small feature numbers.

## Methods

### Dataset

Data for our analyses were obtained from the GDSC database (Release 8.3). More specifically, we downloaded normalized expression values (*Affymetrix Human Genome U219 Array*) for 17 419 genes and drug-screening data in the form of logarithmized IC50 values for all 198 drugs screened in the GDSC2 dataset (*CellTiter-Glo* assay). Out of these 198 drugs, we only considered those 179 drugs for which sensitivity measures for at least 600 cell lines are provided.

Note that the GDSC also provides AUC values as a measure of drug response. However, we found that the concentration ranges used to determine the AUC values do not correspond well to clinically feasible treatment concentrations (cf. Supplementary Figure 12). Consequently, we refrained from their analysis.

### Model inputs and outputs

We trained drug-specific regression models that predict the logarithmized IC50 values of cell lines from their gene expression data using four ML algorithms (random forests, neural networks, boosting trees and elastic net). Model inputs are generated using six FS and three FE techniques that select/compute input features based on normalized gene expression values. Some of the FS methods additionally consider the IC50 values to determine the most informative features. To investigate how the number of input features $k$ affects the model performance, we generated input feature sets for each $k \in \{1,2,3,...,25, 50, 100, 200, 300, 400, 500\}$.

In the following text, we will refer to one *setting* as one combination of ML algorithm, DR technique and number of inputs $k$ used to train a certain model. Details on the investigated ML algorithms and DR techniques are presented below.

### Model training and testing

For each drug, we divided the available cell lines into a training set (80% of cell lines) and a test set (20%). On the training set, we performed a 5-fold CV to determine the best-performing hyperparameters of the ML model (see Table 1) using the mean squared error (MSE) as error measure. For each hyperparameter combination, one final model is trained on the complete training data and its performance is evaluated on the test set.

**Table 1:** Overview of all ML algorithms investigated for the training of models, including the used R/Python packages and tuned hyperparameters. The last column denotes the number of testes hyperparameter combinations. Unless stated otherwise, we employed the default parameters of each algorithm in their respective package. Further information on the architecture and hyperparameters of the trained neural networks can be found in Table 3 of the Supplement

Model	Package	Parameter	Value(s)	#Combinations
Elastic net	glmnet, v. 4.1.3 (R) [49]	alpha	$[0, 1]$ in steps of 0.1	$11 \cdot 20 = 220$
		lambda	$10^{v}$ , $v$: 20 equally spaced values $\in [-2,2]$	
Random forest	ranger, v. 0.13.1 (R) [50]	mtry	$[1,25]$ in steps of 2 and	up to 22
			$[40,200]$ in steps of 20	
Boosting trees	gbm, v. 2.1.8 (R) [51]	n.trees	1-20	$20 \cdot 5 = 100$
		interaction.depth	1-5	
Neural network	Tensorflow, v. 1.13.1	# Hidden layers	1, 2, 3	$3 \cdot 2 \cdot 2 = 12$
	Keras API, v. 2.3.1	Activation function	tanh, ELU (none in output layer)	
	(Python) [52, 53]	Dropout	10%, 30%	

This procedure is performed for each setting (i.e. combination of ML algorithm, DR method and number of inputs) separately. As the training and test data (as well as the data in each CV fold) are identical across all settings for one drug, the performance of different settings can be compared directly.

Note that input features are selected/computed using only samples in the training set (both for the CV and the training of the final model), such that the test data do not influence the choice of features.

### ML algorithms

In the following text, we briefly summarize the four ML algorithms that we compare in this publication. An overview of the used R/Python packages and hyperparameters for each model can be found in Table 1.

The predictor matrix for each model is defined as $X \in \mathbb{R}^{n \times k}$ where each entry $x_{ij}$ corresponds to the value of input feature $j \in \{1,...,k\}$ for cell line $i \in \{1,...,n\}$. Furthermore, $y \in \mathbb{R}^{n}$ denotes the response vector, where each entry $y_{i}$ corresponds to the logarithmized IC50 value of cell line $i$.

#### Elastic net

Elastic net is a regression algorithm that estimates the response $y_{i} \in \mathbb{R}$ of a sample $i$ as a linear combination of its input features $x_{i} = (x_{i1}, \dots , x_{ik}) \in \mathbb{R}^{k}$ [54], i.e. the predicted response $\hat{f}(x_{i})$ is given as 


(1) \begin{align*} \hat{f}(x_{i}) = \beta_{0} + \sum\limits_{j = 1}^{k} \beta_{j} \cdot x_{ij}\end{align*}

The vector $\beta = (\beta _{1},..., \beta _{k}) \in \mathbb{R}^{k}$ contains weights for each feature and is determined such that the squared error between the actual response $y_{i}$ and the predicted response $\hat{f}(x_{i})$ is minimized over all $n$ training samples: 


(2) \begin{align*} &\min_{\beta_{0}, \beta} \sum\limits_{i = 1}^{n} (y_{i} - \hat{f}(x_{i}))^{2} + \lambda \cdot \sum\limits_{j = 1}^{k} \left(\alpha \cdot |\beta_{j}| + (1 - \alpha) \cdot \beta_{j}^{2} \right)\end{align*}

The optimization includes two regularization penalties based on the L1 and L2 norms that shrink feature weights toward zero. The L1 norm allows weights to become exactly zero, such that the respective features are excluded from the model. The parameter $\lambda $ controls how much the regularization impacts the optimization, while $\alpha $ regulates the impact of each norm.

#### Random forests

Random forests are a regression/classification algorithm based on decision trees [55]. They combine predictions of many trees into one prediction through averaging/majority vote. To ensure that the trees differ, they are decorrelated by (1) building each tree on a subset of training samples drawn randomly with replacement and (2) only considering a random subset of features for each decision split in a tree.

#### Boosting trees

Similar to random forests, boosting tree models consist of multiple decision trees. However, they combine predictions of individual trees in an iterative rather than a parallel fashion [56]: in model training, an initial prediction is made as the mean response of all training samples. Next, trees are iteratively added to correct the error of this prediction. Each new tree is trained to estimate the residuals of the prediction obtained by adding up predictions of all previously built trees.

#### Neural networks

Neural networks are prediction models loosely modeled after signal transmission mechanisms in human brains [57]. They consist of multiple layers of nodes (*artificial neurons*) connected through weighted directed edges. Each node receives information through incoming edges, then processes the information, which typically involves applying a non-linear activation function, and passes the resulting value via outgoing edges. There exists a plethora of neural network architectures. Here, we only consider *fully connected*, *feed-forward networks*, where each node is connected to all nodes of the consecutive layer.

### Dimensionality reduction techniques

The GDSC offers expression values for more than 17 000 genes and around 1000 cell lines. This means that the number of features that characterize each sample is considerably larger than the number of samples itself. When performing ML on such a dataset, performing a DR to counteract the *curse of dimensionality* is usually indispensable. Additionally, reducing the input dimension can notably shorten the runtime and computational resources needed for training while increasing model interpretability. In the following text, we introduce the six FS and three FE approaches we investigated in this publication.

#### FS techniques

FS methods aim at choosing a subset of informative features from a given feature set.

**Randomized Feature Selection** 

We generated randomized feature sets by randomly sampling gene sets of size $k$ from all genes with expression values provided in the GDSC. To get a more stable estimate of the prediction errors for random features, we generated 10 random feature sets for each $k$ and averaged the error measures of the 10 corresponding models.

**Literature-based Feature Selection** 

For the literature-based FS, we retrieved a list of cancer driver genes from the IntOGen website (Release 1 February 2020) [58]. We only considered genes for which expression values are provided in the GDSC. Additionally, genes with warnings in the IntOGen database (e.g. genes that are known artifacts) were removed. Next, we sorted the remaining 476 genes according to their smallest IntOGen tier from tier 1 (i.e. genes with the strongest evidence of being cancer drivers) to tier 3. Within each tier, genes were sorted descendingly according to the number of cohorts for which they have been reported as a cancer driver. From the beginning of this sorted gene list, the first $k$ genes were chosen.

**Variance-based Feature Selection** 

For this FS, we chose the $k$ genes, for which the variance of expression values was the largest.

**Correlation-based Feature Selection** 

For this FS, we chose the $k$ genes with the highest absolute Pearson correlation coefficient between the expression values of each gene and the IC50 values of the corresponding cell lines.

**Enrichment-based Feature Selection** 

We developed this FS to identify genes whose up-/downregulation is linked to sensitivity/resistance to a given drug. First, z-scores are calculated for each gene separately, where the mean and standard deviation of expression values were derived from the training data. We then consider a gene upregulated (downregulated) in a cell line if its z-score is larger (smaller) than 1.65, which corresponds to the 95% (5%) percentile of a standard normal distribution.

Our approach uses gene set enrichment analysis (GSEA) [59], as implemented in the GeneTrail webserver [60]. GSEA tests for the accumulation of a certain feature (e.g. up-/downregulation of a gene) at either end of an ordered list of samples (e.g. cell lines): First, cell lines are sorted by increasing IC50 for the drug of interest to obtain a ranked list. Second, a Kolmogorov–Smirnov test [61, 62] is conducted twice per gene to determine whether cell lines in which the gene is (1) up- or (2) downregulated are enriched the top/bottom of the list, respectively. Each test yields a *P*-value and a direction denoting whether the enrichment occurred at the top or bottom of the list. We adjusted the *P*-values using the Benjamini–Hochberg procedure [63] separately for all up- and all downregulated genes. This procedure results in four lists of genes: genes that are up-/downregulated among the most sensitive/resistant cell lines, respectively. To obtain the $k$ most important features, we proceeded as follows: for each list, we order genes from smallest to largest *P*-value and assign a rank to each gene, starting at 1. If a gene occurs in multiple lists, we keep it only in the list with the smallest rank. Next, we merge all lists by sorting genes according to their rank, and *P*-values are used to break ties. From the beginning of this merged list, we then select the first $k$ features.

**MRMR Feature Selection** 

This FS is based on the *minimum-redundancy-maximum-relevance* (MRMR) principle, which aims to select features with a strong dependence on the response variable (i.e. large relevancy) but weak dependence on each other (i.e. small redundancy). Our implementation is based on a greedy heuristic by Kwak and Choi [46] that iteratively selects features, starting with the most informative ones. As dependence-measure, the mutual information $I$ is employed. Let $F$ denote the set of all potential input features (i.e. genes) and $C$ the response variable (i.e. ln(IC50) values for a given drug). Here, both expression and ln(IC50) values were discretized using an equal-width binning with six bins.

Let $S$ be the list of selected features, which is initially empty. In each iteration, the feature $f_{i} \in F$ that maximizes the following term is added to $S$ and removed from $F$: 


(3) \begin{align*} \max_{f_{i} \in F} \; I(C;f_{i}) - \sum_{f_{j} \in S}{ \frac{I(C;f_{j})}{H(f_{j})} \cdot I(f_{i};f_{j})}\end{align*}

Here, $H$ denotes the entropy and $I$ denotes the mutual information. This procedure is repeated until $|S| = k$. The result is a list of $k$ features ordered by importance. To keep the runtime manageable, we limited $F$ to the 1000 genes with the largest mutual information to the IC50s.

As the presented approach is a greedy heuristic, the selected features are not guaranteed to provide an optimal solution to the MRMR problem for a given $k$. Hence, we additionally implemented an MRMR-based FS as a quadratic optimization program (QP). However, the high runtime of this approach only allowed us to compute feature sets for $k\leq 5$ in a reasonable time ($<500$ s for a single $k$ on a single training dataset). Additionally, we found no improvement in test MSE when employing the features selected by the QP instead of the heuristic to train ML models (see Supplement). Consequently, the QP is not discussed further.

#### FE techniques

FE techniques transform a high-dimensional dataset into a lower dimensional representation by generating new features as (non-)linear combinations of the original features. Thereby, information from potentially all features can be condensed into a significantly lower dimension without discarding any features. However, this generally comes with a loss of interpretability regarding the generated features.

**Principal Component Analysis** 

We performed a PCA (R package *stats*, v. 3.6.3) using the expression values of all cell lines in the respective training set to extract a lower dimensional representation of cell lines using the first $k$ principal components. We used the feature coefficients calculated on the training data to project the test set cell lines into the same $k$-dimensional space.

**PASL** 

PASL (Pathway Activity Score Learning) by Karagiannaki *et al*. [40] is a DR approach that aims to produce (biologically) interpretable features. Given a feature matrix (i.e. gene expression data) and predefined feature sets (i.e. genes belonging to a certain pathway), PASL projects the data into a latent space, where each newly constructed feature is a linear combination of features from one of the predefined feature sets. The computed features can be interpreted as pathway activity scores for each sample. Just like PCA, PASL computes features in an ordered manner, such that the features explaining most of the variance in the data are computed first. We applied PASL with default parameters to the training data to generate $k$ pathway features. As feature sets, we considered the same data as Karagiannaki *et al*., namely pathways from KEGG [64], Reactome [65] and BioCarta [66]. Analogously to PCA, we applied the linear combinations computed by PASL to the test cell lines to obtain their representation in the new feature space.

**Autoencoder** 

An autoencoder is a type of neural network that encodes data into a lower dimension. It consists of an encoding part, which generates a lower dimensional representation of the input, and a decoding part, trained to reconstruct the original inputs from the encoded representation. After training the entire network, only the encoder is used to generate the lower dimensional (here: $k$-dimensional) representation of the inputs for training and test data. As one autoencoder has to be trained for each drug, training dataset and $k$ separately, the high runtime (on average 4.5 min for a single model) did not permit us to perform any hyperparameter tuning. Note that tuning would require an additional CV nested inside the main CV described in the Methods section. The used hyperparameters and network architecture can be found in Supplementary Table 4.

## Results

We trained drug-specific models that predict logarithmized IC50 values for 179 drugs of the GDSC2 dataset using the four ML algorithms and nine DR techniques presented above. To investigate how the number of input features $k$ impacts performance, we trained models for each $k \in \{1,2,3,...,25,50,100,200,300,400,500\}$. The hyperparameters of each algorithm (cf. Table 1) were tuned using a 5-fold CV on the training data.

**Figure 1:**
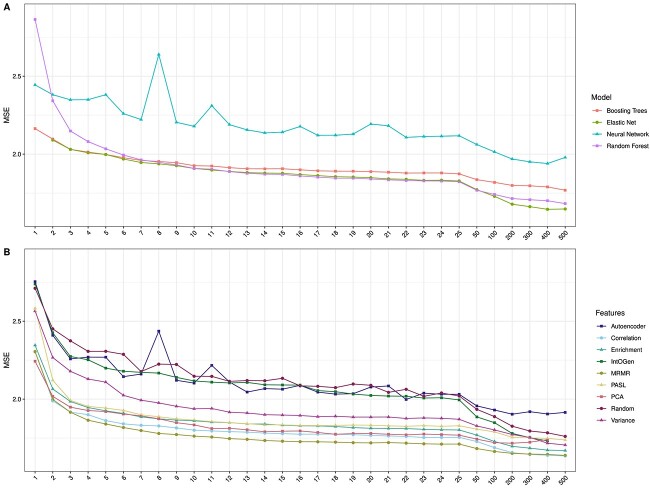
Average test MSEs. This figure depicts the test MSEs averaged over all drugs for each ML algorithm (A) and DR method (B). The x-axis denotes the number of input features, the y-axis denotes the mean test MSE, and the coloring represents the different ML algorithms or DR techniques. Boosting trees, elastic nets and random forests were trained for all 179 drugs in the GDSC2 dataset for which IC50s for more than 600 cell lines were available were. For neural networks, models were only trained on the 50 drugs with most available cell lines due to their large runtime (cf. Supplementary Table 2 and Fig. 5). Additionally, no models could be trained for the following settings: for elastic net, no models for $k=1$ exist since the used R package *glmnet* only allows feature sets with $k\geq 2$. For the IntOGen features, results for $k=500$ do not exist since our filtered IntOGen list (cf. Methods) consists of 476 features only. For the PCA features, results for $k=500$ do not exist since most CV training sets contain less than 500 samples, and the number of principal components computed by the *stats* R package is limited by the number of input samples.

Note that we only trained neural networks on the 50 drugs with the most available cell lines due to their high runtime (cf. Fig. 5 and Supplementary Table 2). Additionally, for a small number of settings, no models could be trained (see Fig. 1).

In the following text, we first analyze the statistical performance of the investigated ML algorithms and DR techniques. Then, we assess the trade-offs between models of different complexity. Next, we show how the prediction of sensitive cell lines can be improved using different error measures for hyperparameter tuning. We then compare the four ML algorithms regarding their runtime and interpretability and exemplarily investigate features derived from the MRMR FS and their importance in elastic nets. Lastly, we show that complex prediction models equally benefit from DR by investigation of a deep learning method by Chiu *et al*. [3].

### Average test MSE

We first analyzed which ML method(s) and DR approach(es) yield the smallest test error across all investigated drugs. Fig. 1A compares the test MSE of all ML models averaged across drugs and DR approaches, while Fig. 1B depicts the test MSE of all DR approaches averaged across drugs and ML models. Overall, errors decrease as $k$ increases. The decrease is most drastic from $k=1$ to $k=10$ and again when $k \geq 50$, especially for elastic net. For $k> 8$, elastic net and random forest yield the smallest MSE, while neural networks consistently perform the worst. A comparison of all algorithms using only the 50 drugs we used to train the neural networks is provided in the Supplement and shows the same trends.

Figure 1B shows that the MRMR and correlation-based FSs, followed by PCA, yield the smallest test MSE. Interestingly, for $k> 200$, the average error of PCA-based models increases. More detailed figures that show results for each combination of ML algorithm and DR method separately are provided in the Supplement. There, it can be seen that using PCA with $k> 200$ only increases errors for boosting trees and random forests but outperforms all other DR methods for elastic net and neural networks.

### Best-performing settings

**Figure 2:**
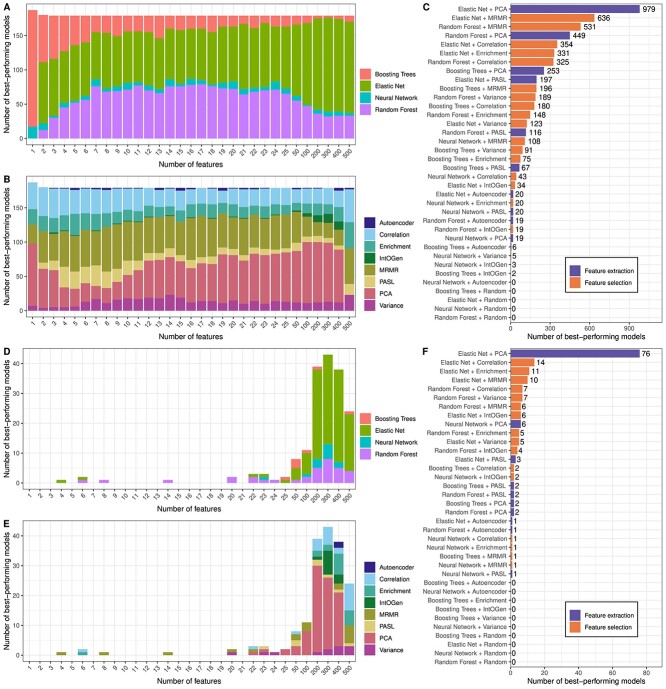
Best-performing models for each drug and number of input features. Sub-figure A and B, respectively, show how often each ML algorithm and DR method yielded the smallest test MSE for each $k$. Sub-figure C shows how often a given combination of ML algorithm and DR method yielded the best results summarized over all $k$. Sub-figures D–F depict the same results, but only the feature number $k$ yielding the smallest test MSE for each drug is shown.

In this section, we analyze which combinations of ML algorithm and DR technique resulted in the smallest test MSE for each drug and how often a certain combination performed best. Figure 2 summarizes how often each combination resulted in the best model for each drug. Sub-figures A to C depict the best-performing combinations for each $k$ and Sub-figures D to F show the same statistics for the best-performing $k$ only.

Figure 2A shows that random forest and elastic net are the most prevalent ML models, with elastic net being more dominant for large feature numbers. Larger feature sets likely contain more redundant or uninformative features, which might be easily ignored by elastic net but not by tree-based models since a random subset of features is selected in each node.

Regarding the DR techniques (cf. Fig. 2B), PCA and MRMR are the most successful, followed by the correlation-based FS. For large $k$, PCA is the most dominant approach. Figure 2C shows how often each combination of ML and DR was the best-performing. In agreement with the previous results, elastic net and PCA are by far the most successful combination.

Sub-figures 2D to F show the same results as A to C but limited to only the $k$ that resulted in the best performance for each drug. For almost all drugs, large feature numbers $\geq 100$ yielded the best results. The combination of elastic net and PCA was the best-performing for 76 of the 179 investigated drugs. However, almost all DR techniques other than the random FS had the best performance for at least some drugs.

**Figure 3:**
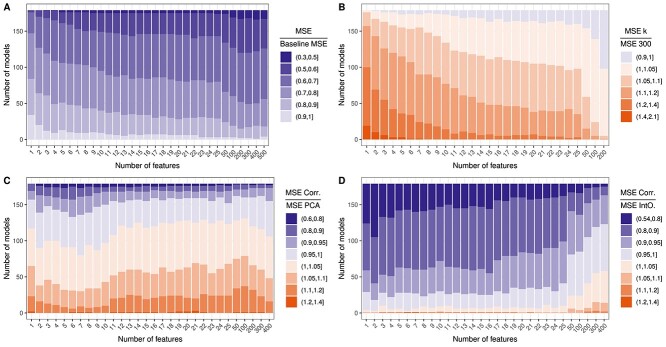
Performance comparison of different models and (numbers of) features. Each sub-figure depicts a comparison of test MSEs for different scenarios, where the MSE for one scenario is divided by the MSE of the other: Sub-figure A compares the test MSE of the best-performing models for each drug and $k$ to the MSE of drug-specific baseline models that always predict the mean ln(IC50) of the training samples. Sub-figure B compares the test MSE of the best-performing models with $k<300$ features to those with $k=300$ features. Sub-figures C and D compare the best-performing models using correlation-based features and features derived from PCA or the IntOGen gene list, respectively.

As discussed previously, FE-based features are inherently difficult to interpret. Therefore, we performed the same analysis using FS algorithms only. The results are presented in Supplementary Figure 7 and show that among all FS approaches, MRMR is chosen most often, followed by the correlation- and enrichment-based approaches. Note that all of these methods utilize not only the expression values but also the IC50 values to derive informative features.

### Baseline

The comparison of test MSEs in the previous sections allowed us to identify the best-performing settings and hyperparameters. However, we do not know whether a model is a good predictor without a proper baseline error.

A straightforward approach to obtain a baseline is to use a *dummy model* that always predicts the mean response (here: mean ln(IC50)) of training samples. Figure 3A depicts the ratio between the test MSE of the best-performing model for each drug and $k$ and the baseline MSE for the corresponding drug. All models are an improvement over the baseline. For 80% of models, the improvement is at least 20% ($\frac{MSE}{Baseline} \leq $ 0.8), and for 18% of models, the improvement is at least 40% ($\frac{MSE}{Baseline} \leq $ 0.6).

In the same manner, models with different input sizes can be compared: For most drugs, the best-performing feature number was around $k=300$ (cf. Fig. 2D) and smaller $k$ resulted in larger errors (cf. Fig. 1). In Fig. 3B, we compare the test MSE of the best-performing model for each drug using $k=300$ features to models with smaller $k$. For most models (63%), the increase in MSE when using $k < 300$ features is rather small (< 10%). For $k=100$, only 3% of models show an error increase of > 10%. For $k=15$, this number rises to 27%, but the model complexity is reduced drastically.

Figure 2 shows that PCA was the best-performing DR method. However, features obtained through FE approaches like PCA are not easily interpretable. In contrast, the correlation-based FS is easy to interpret and implement. In Fig. 3C, we compare the performance of the best-performing models using PCA- and correlation-based features, respectively. For 37% of models, the correlation-based features even performed better than the PCA-based features. For 52% of models, the increase in MSE from using correlation-based features is $<10\%$ and for only 0.4% of models, the increase is $> 20\% $.

In Fig. 3D, we compare the correlation-based FS to the literature-based FS using the IntOGen cancer gene list. The correlation-based features outperform the literature-based ones for 93% of models. Among the 7% of models, where the IntOGen features performed better, improvements were mostly small (< 10% in 94% of models). In summary, these results indicate that using the literature-based over correlation-based FS is not recommended. However, the slight improvements of PCA over correlation might warrant the loss of feature interpretability.

### Improving predictions for sensitive cell lines

A major goal of drug sensitivity prediction is to identify drugs that are effective for a given sample. However, there is a significant under-representation of sensitive samples (i.e. cell lines with small IC50 for a given drug) in drug screening data [5, 11, 16, 21]. Consequently, ML models trained on these data often exhibit a low prediction sensitivity (classification) and a large MSE (regression) for sensitive cell lines [11, 16, 21]. In the following text, we show that using an error measure for model tuning that increases the importance of sensitive cell lines can notably improve their predictions. This approach is similar to using sample weights or upsampling of underrepresented samples (see [5, 11, 16, 21]). To identify samples as *sensitive* or *resistant*, we binarized IC50s for each cell line using drug-specific thresholds as described in [5].

**Figure 4:**
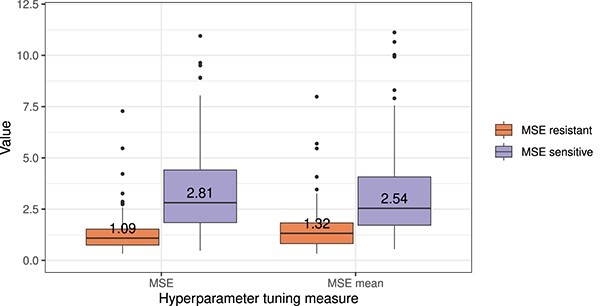
Effect of different hyperparameter tuning measures. This figure compares the test MSEs for sensitive and resistant cell lines when using the conventional MSE for hyperparameter tuning versus the mean between the MSE of all sensitive and all resistant cell lines. To identify a cell line as sensitive/resistant, we compare its ln(IC50) to a drug-specific threshold derived through a procedure described by Knijnenburg *et al*. [5].

Figure 4 depicts the distribution of test MSEs of the best-performing model for each drug selected through CV error. The orange and purple box plots represent the resistant and sensitive cell lines, respectively. The left side of Fig. 4 shows the results when hyperparameters and settings are selected based on CV MSE. For almost all drugs, the test MSE for sensitive cell lines is considerably larger than for resistant ones. For the results shown on the right side of Fig. 4, we did not use the conventional MSE to select the best hyperparameters/setting but instead calculated the MSE of sensitive and resistant cell lines separately and averaged both values. Using this tuning measure, the MSE for sensitive cell lines decreased considerably but remains larger than the MSE for resistant ones.

**Figure 5:**
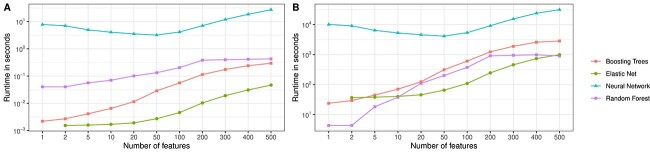
Runtime comparison of ML algorithms. Sub-figure A depicts the mean training duration for a single model (i.e. one hyperparameter combination) for increasing feature numbers. Sub-figure B depicts the duration of performing the 5-fold CV plus fitting of models on the whole training data accumulated over all hyperparameter combinations.

While this approach increases the error of the resistant cell lines, this trade-off might be warranted since predictions for the samples of interest are improved. However, more advanced ML algorithms explicitly focusing on sensitive cell lines, like our previously published method SAURON-RF [16] or RWEN by Basu *et al*. [21], can achieve even larger improvements.

### Runtime

Boosting trees, random forests and elastic nets were trained using 24 cores on an *Intel Xeon Gold 6248* (2.50GHz) CPU. Neural networks were initially trained using a *Nvidia Tesla V100-SXM2* GPU since GPUs are known to effectively parallelize computations needed in network training. However, for comparatively small networks, the overhead of transferring calculations to the GPU can outweigh the computational speedup, which we also observed in our experiments (see Supplementary Figure 8). Hence, we switched from GPU to CPU (*Intel Xeon E5-2698 v4*, 2.20GHz, 24 cores).

Figure 5A depicts the duration of training a single model using each ML algorithm averaged over all trained models. The runtime of neural networks is considerably larger than that of the other algorithms, with elastic net being the fastest. Figure 5B depicts the combined runtime of performing the complete CV and training of final models on all hyperparameter combinations for a given setting. Although we tested the most hyperparameter combinations for elastic nets (cf. Table 1), they remain the fastest approach for $k> 10$.

### Model and feature interpretability

Drug sensitivity prediction aims to provide accurate models but also to identify markers of treatment sensitivity/resistance. However, not all models are equally interpretable. As discussed above, inputs obtained through FS are more interpretable than those obtained through FE. Additionally, ML algorithms exhibit great differences regarding their inherent interpretability. In Table 2, we assess the interpretability of the four investigated ML algorithms based on model *transparency* and *explainability* as described by Lipton [47] and Imrie *et al*. [48]. In an attempt to make models more interpretable, biological knowledge is often explicitly encoded into prediction models, e.g. using pathway-layers in neural networks [4, 35, 67], exploiting known protein interactions [22] or encoding information on known markers of drug response [11]. Li *et al*. found, however, that the explicit incorporation of biological knowledge may decrease model performance and lead to false conclusions [35]. Hence, the assumptions that are introduced by adding biological knowledge to a model should be carefully investigated.

**Table 2:** Interpretability of ML models. We assess the abstract concept of interpretability using the terms *transparency* and *explainability*, as introduced by Lipton [47] and Imrie *et al*. [48], respectively. *Transparency* describes the inherent complexity of an ML model, including the model size in terms of its parameters and the human intelligibility of its computations. *Explainability* focuses on deriving explanations for why certain predictions are obtained for certain inputs. This includes feature importance, the identification of training samples that are similar to a given input, the effect of altering certain inputs and the examination of how certain (human) concepts are interpreted by the model. Notably, inherently non-transparent models (e.g. deep neural networks) can still provide good explainability. However, extracting knowledge from the model might require applying additional tools, which can be challenging, especially for non-ML experts. Here, we only list the explainability methods that are readily available in the used R/Python packages (cf. Table 1). Imrie *et al*. provide a good overview of further tools for different applications and ML algorithms [48]

Model	Transparency	Explainability
Elastic net	**+** easily interpretable feature coefficients	• feature importance: absolute value of coefficients; sign of coefficient denotes impact direction
Random forest	**+** easily interpretable decision splits **–** typically large number of trees	• feature importance: error improvement obtained from splits using certain feature; error increase when feature is randomly perturbed • samples similar to given input: training samples reaching same leaf nodes
Boosting trees	**+** easily interpretable decision splits **–** typically large number of trees **–** trees affect predictions to varying degree	• feature importance: error improvement obtained from splits using certain feature; error increase when feature is randomly perturbed
Neural network	**–** typically thousands of model parameters **–** complex, multi-layered computations to offset inputs against each other	

**Figure 6:**
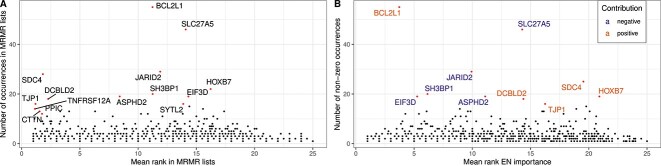
Feature importance of MRMR features. Sub-figure A shows the importance of features selected by the MRMR FS with $k=25$ for all drugs. The x-axis denotes the mean rank of each gene in the feature lists of all drugs, and the y-axis denotes the number of drug-lists in which the gene occurs. Sub-figure B shows the importance of the MRMR features in the best-performing elastic net models trained on these features. The x-axis denotes the mean rank of each gene according to the absolute size of feature coefficients derived from the trained models, and the y-axis denotes the number of models in which the feature had a non-zero coefficient. The color denotes whether the genes have a negative/positive coefficient (cf. $\beta _{j}$ in Equation 1) in the trained models, indicating their tendency to de-/increase predicted IC50s.

However, training an ML model is not always necessary to identify features that impact drug response: Three of the investigated FS approaches (Correlation, Enrichment, MRMR) not only consider the expression data but also the drug response values. Consequently, the chosen features already provide information on potential markers of sensitivity/resistance. Since we found that MRMR is the best-performing FS method, we investigated the selected features more closely: Figure 6A depicts how often each gene was chosen by the MRMR FS for $k = 25$ across all drugs and its average rank in the drug-specific MRMR lists. Features that are chosen often, such as BCL2L1 and SLC27A5, might be interesting biomarkers across drugs. Indeed, BCL2L1 expression was shown to prevent apoptosis, thereby conferring multi-drug resistance to cancer cell lines [68]. Increased apoptosis and drug sensitivity were observed when BCL2L1 was silenced or inhibited [69–71]. Gao *et al*. [72] found that SLC27A5 deficiency was related to poor prognosis, proliferation and drug resistance in hepatocellular carcinoma. Knockout of SLC27A5 activates the KEAP1/NRF2 pathway [72], which is linked to chemoresistance in non-small cell lung cancer patients [73] and different types of cancer cell lines [74]. In contrast, features rarely chosen by MRMR but with a small rank are likely important for specific drugs only and can be assessed to study drug-specific mechanisms of treatment response.

We next investigated whether the features that are selected often by MRMR are also the ones with the highest impact in elastic net models trained with those features. We chose elastic nets for this analysis due to the straightforward interpretation of their coefficients (cf. $\beta $ in Equation 1). Figure 6B depicts how often the selected MRMR features had a nonzero contribution in the trained elastic nets and their mean rank based on the absolute size of assigned coefficients. Again, BCL2L1 and SLC27A5 were impactful features for a large number of drugs (55 and 46 drugs each). The sign of the coefficients indicates whether increasing the expression of a gene positively or negatively affects the predicted IC50s. For 98% of genes, their contribution was either always positive or always negative for at least 99% of drugs, indicating that features generally have a consistent impact on drug response.

### Impact of DR on a multi-omics multi-drug deep learning model

Our benchmarking mainly focuses on predicting drug response based on gene expression features using standard ML algorithms. However, state-of-the-art prediction models exhibit increasingly complex architectures and are often based on multi-omics characterizations of cell lines, sometimes including also molecular characterizations of drugs [75]. Hence, we investigated the effect of different DR methods on a multi-omics multi-drug deep learning model by Chiu *et al*. [3] and compared its performance with that of elastic nets and random forests. Their approach uses gene expression and mutation data as input, which are projected into a lower dimension ($k = 64$, each) using autoencoders. The encoders are connected to a deep neural network, with drug-specific output nodes predicting each drug’s IC50. To conform to our analysis setup, we slightly modified the autoencoder pre-training. For an in-depth description of the model and our performed analyses, please refer to the Supplement. The results can be summarized as follows:

- The model by Chiu *et al*. is outperformed by both drug-specific elastic net and random forest models using $k = 64$ correlation-based expression features for all 170 investigated drugs (cf. Supplementary Figure 10A, B).
- Even when the autoencoders by Chiu *et al*. are trained to generate larger feature embeddings of as many as $k = 500$ features for each omics type, they are outperformed by drug-specific elastic net and random forest models using only $k \leq 10$ features for 95% and 90% of drugs, respectively.
- When replacing the autoencoders in Chiu *et al*.’s approach with PCA or correlation-based features, the performance is improved for 83% and 76% of models, respectively (cf. Supplementary Figures 10C, D).
- Expression features outperformed mutation features for almost all investigated models and feature numbers (cf. Supplementary Figures 9 and 11).

In summary, this exemplary analysis highlights that single-drug models with small feature numbers can outperform more complex multi-drug and multi-omics approaches. It also proves again that the choice of ML/DR method substantially impacts predictions.

## Conclusion

We performed a comprehensive analysis of the prediction of IC50 values using four ML algorithms in combination with six FS and three FE techniques. Our evaluations on the GDSC2 dataset show that elastic nets using features obtained through PCA yielded the smallest test MSE for 76 of 179 investigated drugs. Elastic nets also showed the lowest runtime and allow a straightforward identification of features with a strong impact on predictions. In contrast, neural networks including the more sophisticated deep learning approach by Chiu *et al*. [3] had the worst performance. This aligns well with findings by Li *et al*. [35].

Among the FS methods, the MRMR-based approach performed best. In general, FS methods considering the drug response performed better than methods using only expression values. Methods that do not consider either drug response or gene expression, like the literature-based or random FS, performed worst. However, on a dataset other than the GDSC, features like known cancer genes might yield more robust predictions than features tailored to a specific dataset.

Our analyses focused mainly on gene expression data since it was shown to be the most informative data type for drug sensitivity prediction [3, 39, 42]. Our analyses using mutation data (cf. Supplementary Figure 11) agree with these findings. However, combining different omics data types might prove beneficial, especially when small feature sets are desired. There are also several ML approaches that employ not only cell line features but also drug features in the form of molecular fingerprints [7, 9, 10, 15, 19, 20, 24, 27, 29, 31, 33] (see also [75] for a thorough review). Here, FS could provide insight into which drug properties impact treatment response.

Our analyses primarily focused on training models for each drug separately. However, multi-drug models such as the multi-task network by Chiu *et al*. [3] enjoy popularity. Our analyses show that single-drug models can outperform multi-drug models and might, thus, be preferred when predicting the drug response for an unknown sample (cell line) to a known drug. For an unknown drug, some multi-drug models can directly be applied given that a representation of the drug of interest, e.g. a molecular fingerprint, is available. In contrast, single-drug models are not directly applicable. Nevertheless, molecular fingerprints could be used to identify drugs with available models that are structurally similar to the unknown drug (cf. [13]). The corresponding single-drug models could then be combined into an ensemble model. Whether such an ensemble model would outperform conventional multi-drug models for unknown drugs has yet to be determined. However, we would like to emphasize specific challenges that occur when training any model that should predict responses for multiple drugs:

- It must be ensured that predictions are not primarily driven by drugs with the most available training data.
- The used sensitivity measure should be comparable across drugs, which does not apply to common measures such as IC50 or AUC (cf. Lenhof and Eckhart *et al*. [17], where we suggest a novel measure with across drug comparability). Otherwise, evaluation metrics will be artificially inflated/deflated.
- The training and test sets must be generated so that data leakage is prevented, i.e. no cell line is in the training set of one drug while being in the test set of another drug.

While we focused on monotherapy prediction, drug synergies are increasingly studied using ML to predict the efficacy of drug combinations [76] since combination therapy is common in cancer treatment. In this context, FS could identify features that enhance or impede the interplay between compounds.

Interpretability and trust in predictions are crucial when ML models should eventually be used for clinical decision support. Through performance comparisons with the deep learning multi-omics multi-drug approach by Chiu *et al*. [3], we showed that interpretable models with small feature numbers can substantially outperform complex prediction algorithms. Moreover, our results indicate that complex models equally benefit from using simple DR methods.

Overall, we believe that the methods and evaluation strategies we discussed are helpful tools for the development and assessment of both simple and advanced models for drug sensitivity prediction, independent of the specific algorithms and features at hand.

**Key Points**

- We conducted a large-scale performance comparison and runtime benchmarking of four ML methods and nine DR techniques applied to 179 anti-cancer drugs from the GDSC database resulting in over 16 million investigated models.
- The choice of ML/DR methods strongly impacts prediction performance for both basic and complex ML models.
- Standard models with small feature numbers can substantially outperform complex models.
- Elastic net models result in the best predictions for most drugs while exhibiting the lowest runtime and being easy to interpret.
- PCA performs best among the investigated DR approaches, but correlation-based feature selection is competitive and yields more interpretable features.

## Supplementary Material

Supplement_Benchmarking_ML_DR_Methods_bbae242

## Data Availability

Cell line data were downloaded from the GDSC website (Release 8.3, https://www.cancerrxgene.org/downloads/bulk_download). Our implementations are available at https://github.com/unisb-bioinf/ML_DR_Benchmarking_Drug_Sensitivity_Prediction.

## Author Biographies

**Lea Eckhart** and **Kerstin Lenhof** are PhD students at the chair of **Prof. Dr. Hans-Peter Lenhof**, *Center for Bioinformatics* at *Saarland Informatics Campus of Saarland University*, Germany.

**Lisa-Marie Rolli** is a student research assistant at the chair. Their research focuses on developing advanced and reliable machine learning models for drug sensitivity prediction in cancer.
