# Supplementary material for: A comprehensive benchmarking of machine learning algorithms and dimensionality reduction methods for drug sensitivity prediction
Source: Brief Bioinform. 2024 May 26;25(4):bbae242. doi: 10.1093/bib/bbae242 (PMC11128483; doi:10.1093/bib/bbae242)
Supplement: Supplement_Benchmarking_ML_DR_Methods_bbae242 [file supplement_benchmarking_ml_dr_methods_bbae242.pdf]

# - Supplement -
A comprehensive benchmarking of machine learning algorithms and dimensionality reduction methods for drug sensitivity prediction

Lea Eckhart, Kerstin Lenhof, Lisa-Marie Rolli and Hans-Peter Lenhof

1 MRMR Quadratic Program

In the main manuscript we present a feature selection based on the *minimum-redundancy-maximum-relevance* (MRMR) principle [33]. As the presented approach is a greedy heuristic, it is not guaranteed that the selected features provide an optimal solution to the MRMR problem for a given feature number k. Hence, we additionally implemented an MRMR-based feature selection as a quadratic optimization program (QP). Let F denote the set of all potential input features and C the response variable. Furthermore, let k be the number of features to be selected. To measure the dependence between two variables, the mutual information I is used. Since both, gene expression and IC50 values, are continuous, they must be discretized to calculate their mutual information. To this end, we applied an equal width binning to partition the samples of each feature and each drug response variable into six bins.
The QP can be described as follows: For each feature $f_i \in F$, let x_i be a binary variable that denotes whether f_i is selected ($x_i = 1$) or not ($x_i = 0$). The optimization then selects k features such that the mutual information between features and response is maximized, while the mutual information between selected features is minimized:

$$\max_x \sum_{i=1}^{|F|} x_i \cdot I(C; f_i) - \left(\frac{1}{2} \cdot \sum_{j \neq i} x_i \cdot x_j \cdot \frac{I(C; f_j)}{H(f_j)} \cdot I(f_i; f_j) \right), \tag{1}$$

$$\text{such that } \sum_{i=1}^{|F|} x_i = k \tag{2}$$

Here, $I(a; b)$ denotes the mutual information of two vectors a and b and $H(a)$ denotes the entropy of a.
The ILP was solved using the IBM ILOG CPLEX Optimization Studio V12.6.2 for C++ using 32 cores on an *Intel Xeon Gold 6248* (2.50GHz) CPU. To keep runtime manageable, we limited F to the set of 100 genes for which the mutual information to the investigated drug was largest. Still, we were only able to compute feature sets with $k > 5$ features for a subset of drugs and could not compute any sets for $k > 10$ in a reasonable time (< 500 seconds for a single k on a single training dataset). Figure 1 shows a performance comparison of the greedy heuristic and the QP.

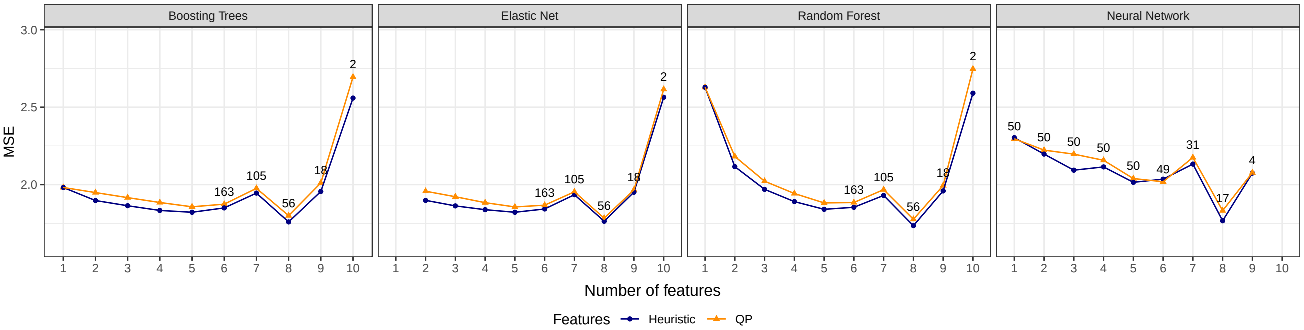

Figure 1: Comparison of QP and heuristic for MRMR feature selection. The average test MSE over all drugs for the best model (i.e., the one with smallest CV MSE) using the respective ML algorithm, feature selection and number of features is shown. As the QP-based features could only be computed for a subset of drugs in the predefined time (< 500 seconds for a single k on a single training dataset), results are only shown for this subset of drugs. Where the number of drugs was smaller than the complete dataset (179 drugs), the data is labeled with the number of drugs over which the average MSE was computed.

Table 1: Overview of different machine learning publications in the field of drug sensitivity prediction. This table provides a brief characterization of the methodology of each approach and lists the dimension reduction (DR) technique that was applied to derive cell line-based input features. Additionally, it is denoted whether a performance comparison of features obtained through different DR methods was performed in each publication. Table continues on next page.

Publication	Methodology	DR for cell line features	DR comparison
Deng et al. (2020) [1]	deep neural network with pathway-layer	literature-based	none
LOBICO by Knijnenburg et al. (2016) [2]	integer linear program	literature-based	none
Menden et al. (2013) [3]	neural network	literature-based	none
MERIDA by Lenhof et al. (2021) [4]	integer linear program	literature-based	none
CDRscan by Chang et al. (2018) [5]	convolutional neural network	literature-based	none
PPORank by Liu et al. (2022) [6]	reinforcement learning with deep neural network	literature-based	none
Stanfield et al. (2017) [7]	proximity networks with random walk	literature-based (FATHMM)	none
Dr.VAE by Rampášek et al. (2019) [8]	variational autoencoder	literature-based, autoencoder	none
QRF by Fang et al. (2018) [9]	quantile regression forest	correlation, random forest feature importance	none
SAURON-RF by Lenhof et al. (2022) [10]	joint classification and regression random forest	min.-redundancy-max.-relevance	none
Lenhof, Eckhart et al. (2023) [11]	conformal prediction using joint classification and regression random forest	min.-redundancy-max.-relevance	none
Matlock et al. (2018) [12]	model stacking	RELIEFF	none
HARF by Rahman et al. (2017) [13]	random forest	RELIEFF	none
Deep-Resp-Forest by Su et al. (2019) [14]	deep cascaded forest	random	none
RWEN by Basu et al. (2018) [15]	iteratively weighted elastic net	elastic net	none
NetBiTE by Oskooei et al. (2019) [16]	biased tree ensemble	feature biasing	none
DeepDR by Chiu et al. (2019) [17]	deep neural network with two autoencoders	autoencoder	PCA
SRMF by Wang et al. (2017) [18]	similarity-regularized matrix factorization	matrix factorization	none
PathDSP by Tang et al. (2021) [19]	deep neural network	pathway enrichment	none
Zhang et al. (2015) [20]	drug and cell similarity network	none	none
NCFGER by Liu et al. (2018) [21]	neighbor-based collaborative filtering	none	none
HNMDRP Zhang et al. (2018) [22]	cell, drug, and drug target similarity network	none	none
ADRML by Ahmadi et al. (2020) [23]	manifold learning with cell line and drug similarity	none	none

Continuation of Table 1.

Publication	Methodology	DR for cell line features	DR comparison
KRL by He et al. (2018) [24]	kernelized rank learning	none	PCA
Rahman and Pal (2016) [25]	(multivariate) random forest	unknown	none
GraphDRP by Nguyen et al. (2022) [26]	graph convolutional neural network	unknown	none
GraphCDR by Liu et al. (2021) [27]	graph neural network with contrastive learning	literature-based, late integration network embedding*	none
RAMP by Lee et al. (2022) [28]	Bayesian neural network with contrastive regularization	network embedding*	none
MOLI by Sharifi-Noghabi et al. (2019) [29]	multi-omics late integration deep neural network	variance-based, late integration network embedding*	early integration embedding*
NeRD by Cheng et al. (2022) [30]	multi-omics neural network	autoencoder, late integration network embedding*	none
GADRP by Wang et al. (2023) [31]	graph convolutional network	autoencoder	PCA
mVAEN by Jia et al. (2023) [32]	elastic net	variational autoencoder	PCA, autoencoder

* These approaches use neural networks to derive a lower-dimensional representation of multi-omics cell line features. We listed this as a type of dimension reduction, since it can be seen as an embedded feature extraction. However, based on this definition, any neural network can be interpreted as performing feature extraction, since each hidden layer is technically a lower-dimensional representations of the input features.

Table 2: Overview of all investigated drugs from the GDSC2 dataset with available IC50s for at least 600 cell lines. Only the 50 drugs with most cell lines were used to train neural networks. Table continues on next page.

	Drug name	ID	# Cell lines
1	Camptothecin	1003	808
2	5-Fluorouracil	1073	806
3	Afatinib	1032	805
4	Taselisib	1561	805
5	PD0325901	1060	804
6	Linsitinib	1510	804
7	Sapitinib	1549	804
8	Luminespib	1559	804
9	Alpelisib	1560	804
10	SCH772984	1564	804
11	LGK974	1598	804
12	Oxaliplatin	1089	802
13	Irinotecan	1088	801
14	GSK1904529A	1093	801
15	EPZ004777	1237	801
16	EPZ5676	1563	801
17	PLX-4720	1036	797
18	Staurosporine	1034	773
19	Nutlin-3a (-)	1047	773
20	MG-132	1862	773
21	MK-2206	1053	771
22	Trametinib	1372	771
23	Palbociclib	1054	770
24	MK-1775	1179	770
25	Cisplatin	1005	768
26	Docetaxel	1007	766
27	Pictilisib	1058	766
28	AZD7762	1022	764
29	Fulvestrant	1200	764
30	Olaparib	1017	762
31	Dasatinib	1079	760
32	AZD3759	1915	760
33	Vorinostat	1012	758
34	PD173074	1049	758
35	Nilotinib	1013	757
36	Paclitaxel	1080	757
37	Sorafenib	1085	757
38	Dabrafenib	1373	757
39	Lapatinib	1558	757
40	AZD4547	1786	757
41	Gemcitabine	1190	756
42	Bortezomib	1191	756
43	Tamoxifen	1199	756
44	Venetoclax	1909	756
45	Wee1 Inhibitor	1046	755
46	Cytarabine	1006	752
47	Gefitinib	1010	752
48	Dactolisib	1057	752
49	BMS-536924	1091	752
50	Erlotinib	1168	752

	Drug name	ID	# Cell lines
51	YK-4-279	1239	752
52	Epirubicin	1511	752
53	BDP-00009066	1866	752
54	Buparlisib	1873	752
55	Ulixertinib	1908	752
56	AGI-5198	1913	752
57	AZD5363	1916	752
58	AZD6738	1917	752
59	AZD8186	1918	752
60	Osimertinib	1919	752
61	Cediranib	1922	752
62	Ipatasertib	1924	752
63	GDC0810	1925	752
64	GSK2578215A	1927	752
65	I-BRD9	1928	752
66	Telomerase Inhibitor IX	1930	752
67	NVP-ADW742	1932	752
68	P22077	1933	752
69	UMI-77	1939	752
70	Sepantronium bromide	1941	752
71	MIM1	1996	752
72	WEHI-539	1997	752
73	BPD-00008900	1998	752
74	Navitoclax	1011	751
75	Cyclophosphamide	1512	751
76	ABT737	1910	751
77	Afuresertib	1912	751
78	MIRA-1	1931	751
79	Savolitinib	1936	751
80	WIKI4	1940	751
81	Vinblastine	1004	750
82	Temozolomide	1375	750
83	Pevonedistat	1529	750
84	Foretinib	2040	750
85	Pyridostatin	2044	750
86	Vinorelbine	2048	750
87	Ulixertinib	2047	749
88	BIBR-1532	2043	749
89	MK-8776	2046	749
90	Talazoparib	1259	748
91	AMG-319	2045	747
92	VX-11e	2096	746
93	LJI308	2107	746
94	AZ6102	2109	746
95	Rapamycin	1084	745
96	Uprosertib	2106	745
97	GSK591	2110	745
98	AT13148	2170	745
99	VE821	2111	744
100	Dactinomycin	1911	740

Continuation of Table 2.

	Drug name	ID	# Cell lines
101	GNE-317	1926	738
102	Crizotinib	1083	737
103	Uprosertib	1553	735
104	Entinostat	1593	735
105	Alisertib	1051	730
106	Mirin	1048	728
107	Obatoclax Mesylate	1068	728
108	Oxaliplatin	1806	728
109	PRIMA-1MET	1131	728
110	Niraparib	1177	728
111	Fulvestrant	1816	728
112	BMS-345541	1249	728
113	XAV939	1268	728
114	AZD5438	1401	728
115	AZD2014	1441	728
116	AZD1332	1463	728
117	Ruxolitinib	1507	728
118	Leflunomide	1578	728
119	VE-822	1613	728
120	WZ4003	1614	728
121	CZC24832	1615	728
122	PFI3	1620	728
123	PCI-34051	1621	728
124	Wnt-C59	1622	728
125	OTX015	1626	728
126	ML323	1629	728
127	Entospletinib	1630	728
128	PRT062607	1631	728
129	AGI-6780	1634	728
130	Picolinici-acid	1635	728
131	ERK_2440	1713	728
132	ERK_6604	1714	728
133	IRAK4_4710	1716	728
134	JAK1_8709	1718	728
135	VSP34_8731	1734	728
136	Selumetinib	1736	728
137	JAK_8517	1739	728
138	Zoledronate	1802	728
139	Acetalax	1804	728
140	Carmustine	1807	728
141	Topotecan	1808	728
142	Teniposide	1809	728
143	Mitoxantrone	1810	728
144	Dactinomycin	1811	728
145	Fludarabine	1813	728
146	Podophyllotoxin bromide	1825	728
147	Gallibiscoquinazole	1830	728
148	Elephantin	1835	728
149	Sinularin	1838	728
150	LY2109761	1852	728
151	OF-1	1853	728
152	MN-64	1854	728
153	KRAS (G12C) Inhibitor-12	1855	728
154	Dinaciclib	1180	727
155	AZD1208	1449	727
156	LCL161	1557	727
157	IWP-2	1576	727
158	I-BET-762	1624	727
159	RVX-208	1625	727
160	GSK343	1627	727
161	AZD5153	1706	727
162	CDK9_5576	1708	727
163	CDK9_5038	1709	727
164	PAK_5339	1730	727
165	TAF1_5496	1732	727
166	IGF1R_3801	1738	727
167	Nelarabine	1814	727
168	ULK1_4989	1733	726
169	Dihydrorotenone	1827	726
170	Sabutoclax	1849	726
171	AZ960	1250	725
172	IAP_5620	1428	725
173	Eg5_9814	1712	724
174	AZD5991	1720	724
175	Ibrutinib	1799	724
176	Vincristine	1818	722
177	GSK2606414	1618	721
178	AZD5582	1617	716
179	Docetaxel	1819	669

Table 3: Overview of all hyperparameters that were investigated for the training of neural networks.

Parameter	Value(s)
Loss function	MSE
Optimizer	Adam
Learning rate	0.001 (default)
# Hidden layers	1, 2, 3
# Nodes per layer	input: k, output: 1, hidden: evenly spaced between in- and output
Activation function	tanh, ELU (none in output layer)
Weight initialization	Glorot uniform for tanh activation, He normal for ELU
Bias initialization	0.01
Weight regularization	L2
Bias regularization	none (default)
Dropout	10%, 30%
Batch size	64
Epochs	max. 4000 (early stopping using 20% of samples as validation data)
Patience	15 epochs

Table 4: Overview of all hyperparameters that were used for the training of autoencoders.

Parameter	Value(s)
Loss function	MSE
Optimizer	Adam
Learning rate	0.001 (default for Adam)
# Nodes per layer	input: 17,419; hidden (encoder): 3,484 and 697; bottleneck: k;<br>hidden (decoder): 697 and 3,484; output: 17,419
Activation function	RELU (none in last encoder layer)
Weight initialization	Glorot uniform (default)
Bias initialization	0 (default)
Weight regularization	none (default)
Bias regularization	none (default)
Dropout	none (default)
Batch size	64
Epochs	max. 100 (early stopping using 20% of samples as validation data)
Patience	5 epochs

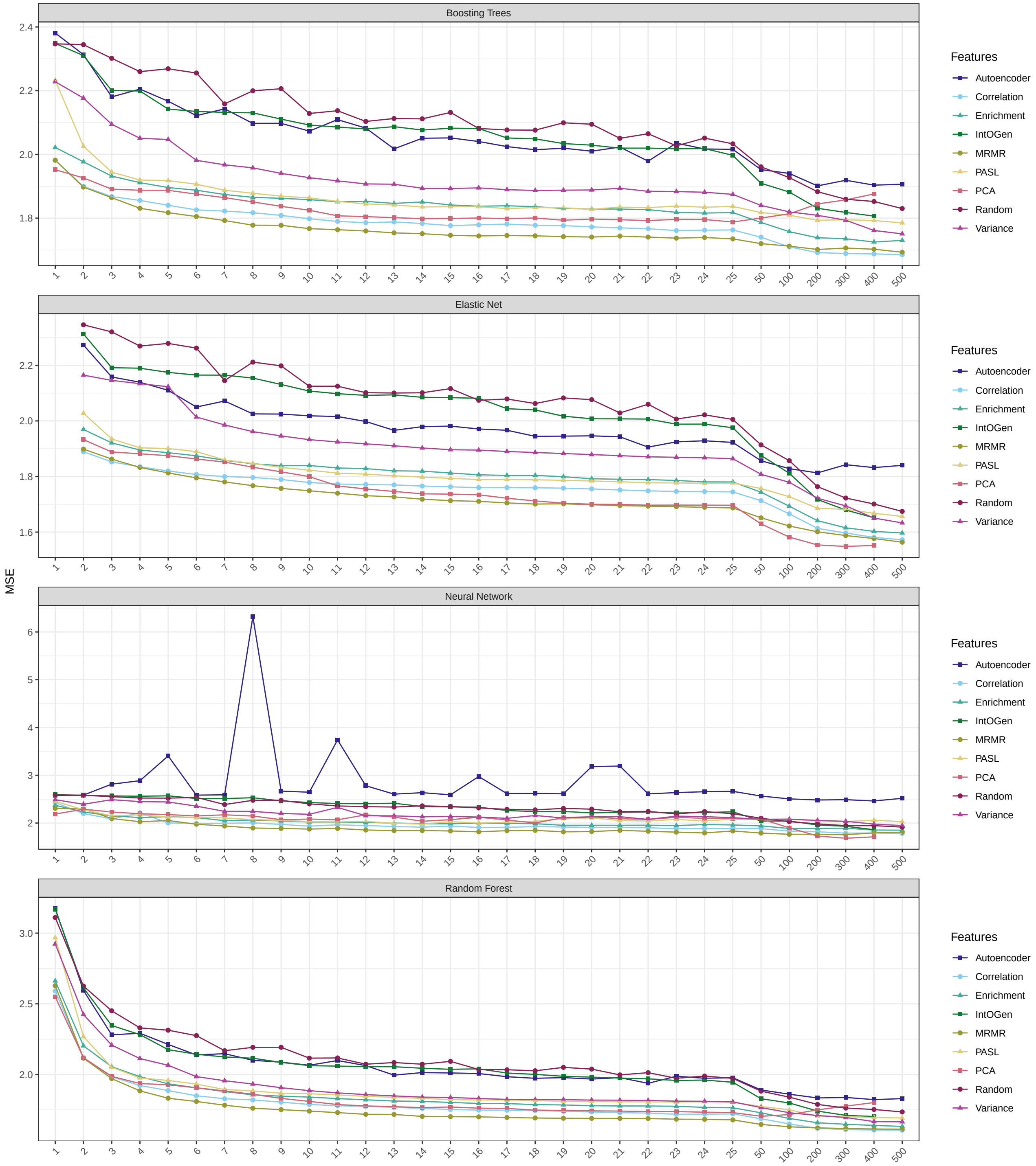

Figure 2: Average test MSEs for each ML method. This figure depicts the test MSEs averaged over all drugs for each combination of DR algorithm, ML method and number of input features. Each plot corresponds to one ML method, where the x-axis denotes the number of input features, the y-axis denotes the mean test MSE and the coloring represents the different DR techniques. Boosting trees, elastic nets and random forests were applied to all 179 drugs in the GDSC2 dataset for which IC50s for more than 600 cell lines were available. For neural networks, models were only trained on the 50 drugs with most available cell lines (c.f. Figure 2).

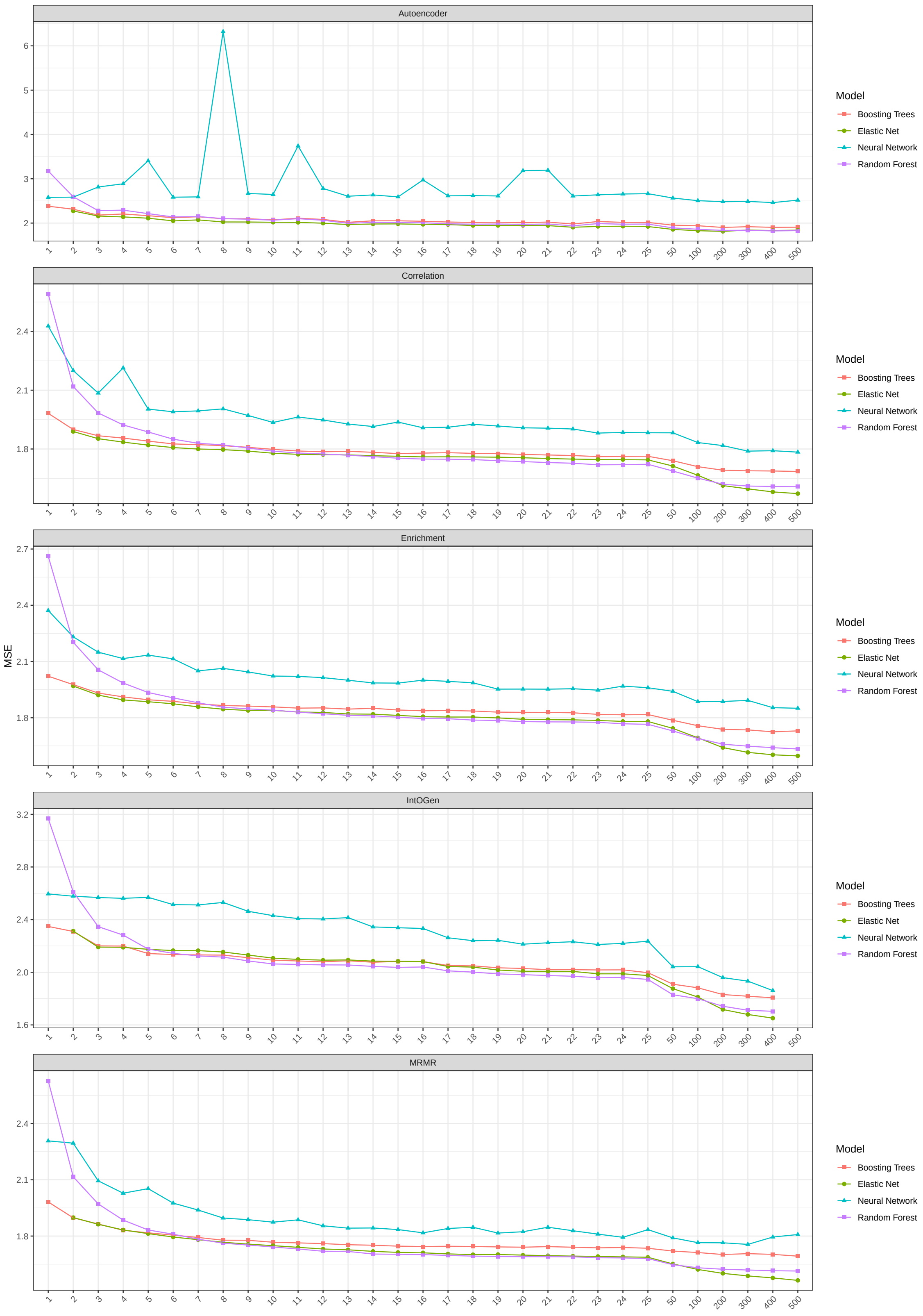

Figure 3: Figure continues on next page.

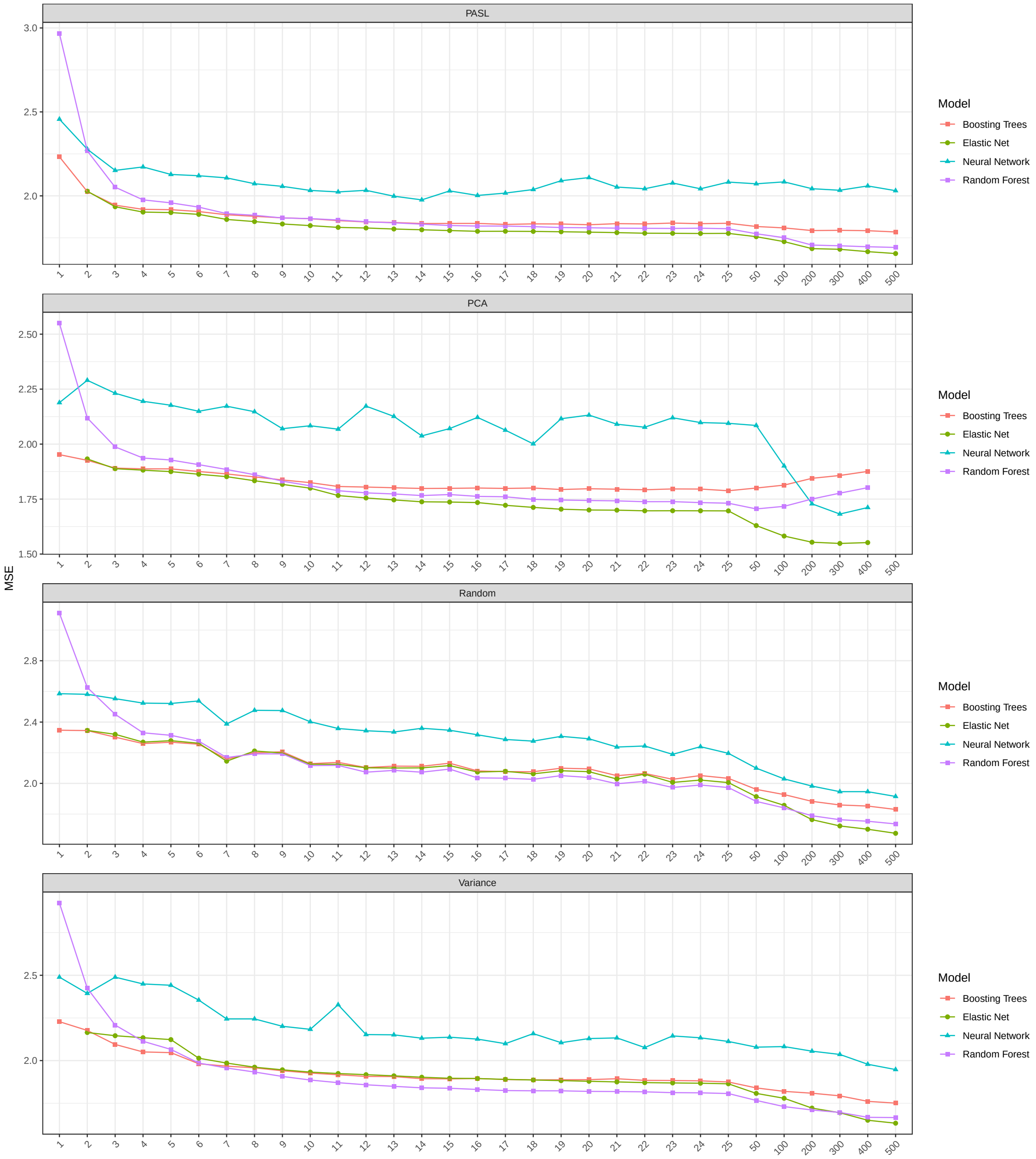

Figure 3: Average test MSEs for each DR algorithm. This figure depicts the test MSEs averaged over all drugs for each combination of DR algorithm, ML method and number of input features. Each plot corresponds to one DR algorithm, where the x-axis denotes the number of input features, the y-axis denotes the mean test MSE and the coloring represents the different ML methods. Boosting trees, elastic nets and random forests were applied to all 179 drugs in the GDSC2 dataset for which IC50s for more than 600 cell lines were available. For neural networks, models were only trained on the 50 drugs with most available cell lines (c.f. Figure 2).

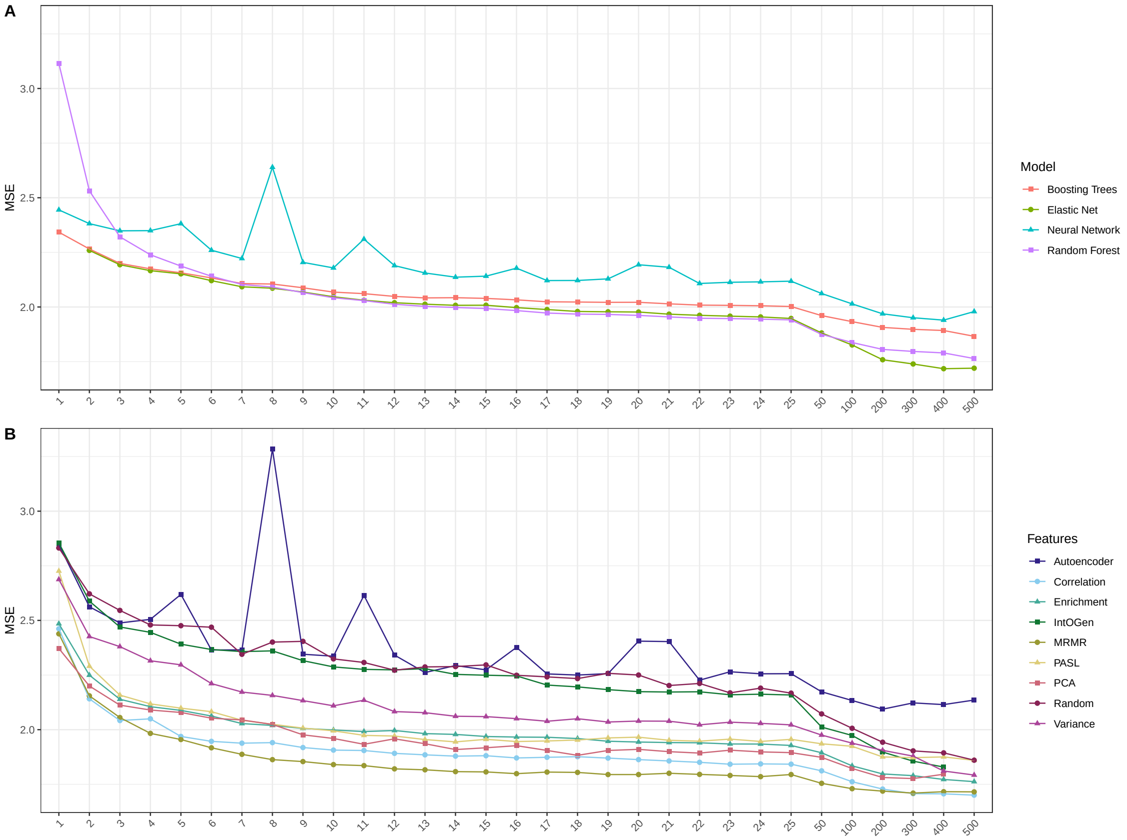

Figure 4: Average test MSEs for the 50 drugs with most cell lines. This figure depicts the test MSEs averaged over the 50 drugs with most cell lines (c.f. Table 2) for each ML algorithm (A) and DR method (B). The x-axis denotes the number of input features, the y-axis denotes the mean test MSE and the coloring represents the different ML algorithms or DR techniques. Boosting trees, elastic nets and random forests were applied to all 179 drugs in the GDSC2 dataset for which IC50s for more than 600 cell lines were available were. For neural networks, models were only trained on the 50 drugs with most available cell lines.

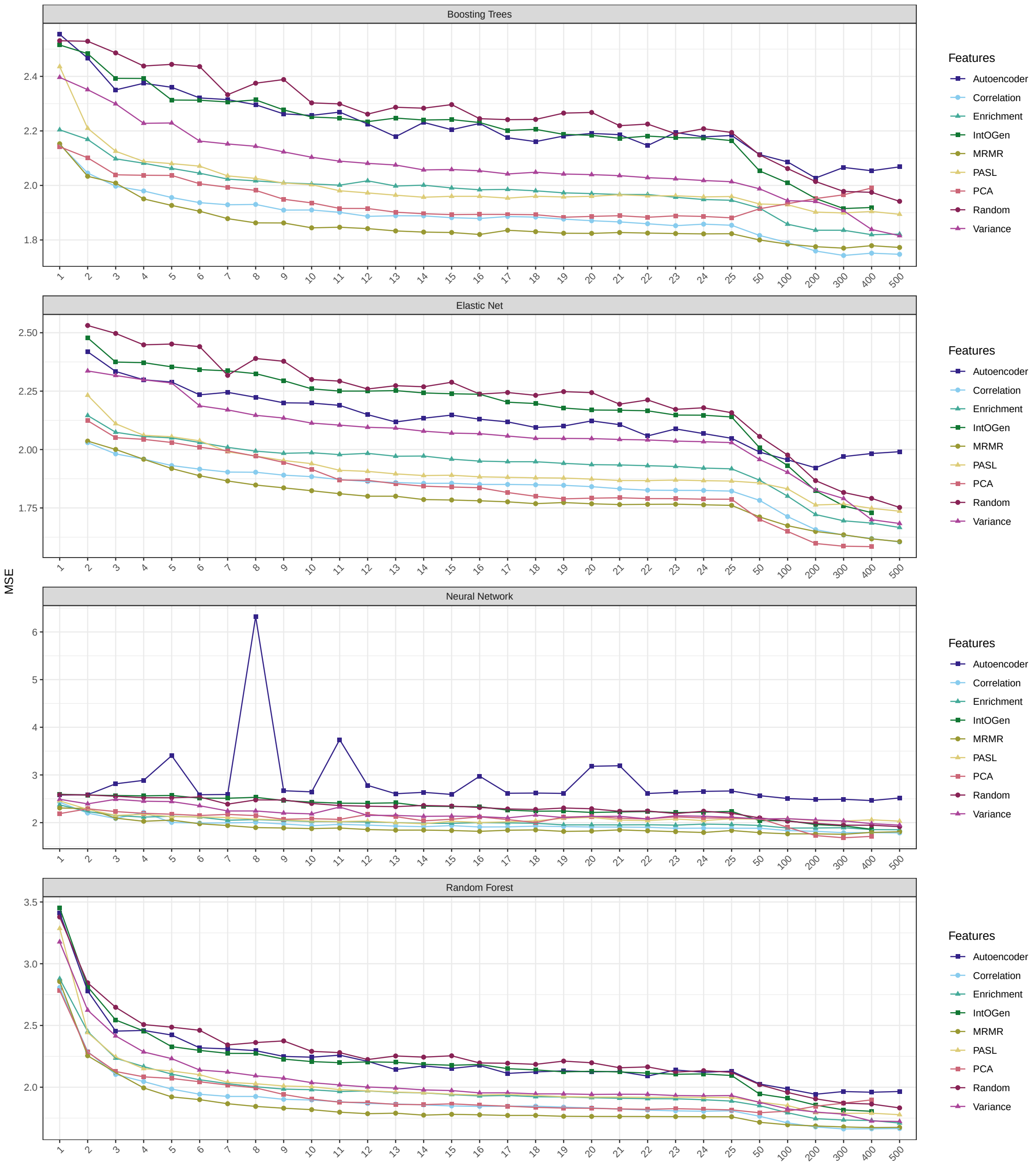

Figure 5: Average test MSEs for each ML method for 50 drugs with most cell lines. This figure depicts the test MSEs averaged over the 50 drugs with most cell lines (c.f. Table 2) for each combination of DR algorithm, ML method and number of input features. Each plot corresponds to one ML method, where the x-axis denotes the number of input features, the y-axis denotes the mean test MSE and the coloring represents the different DR techniques.

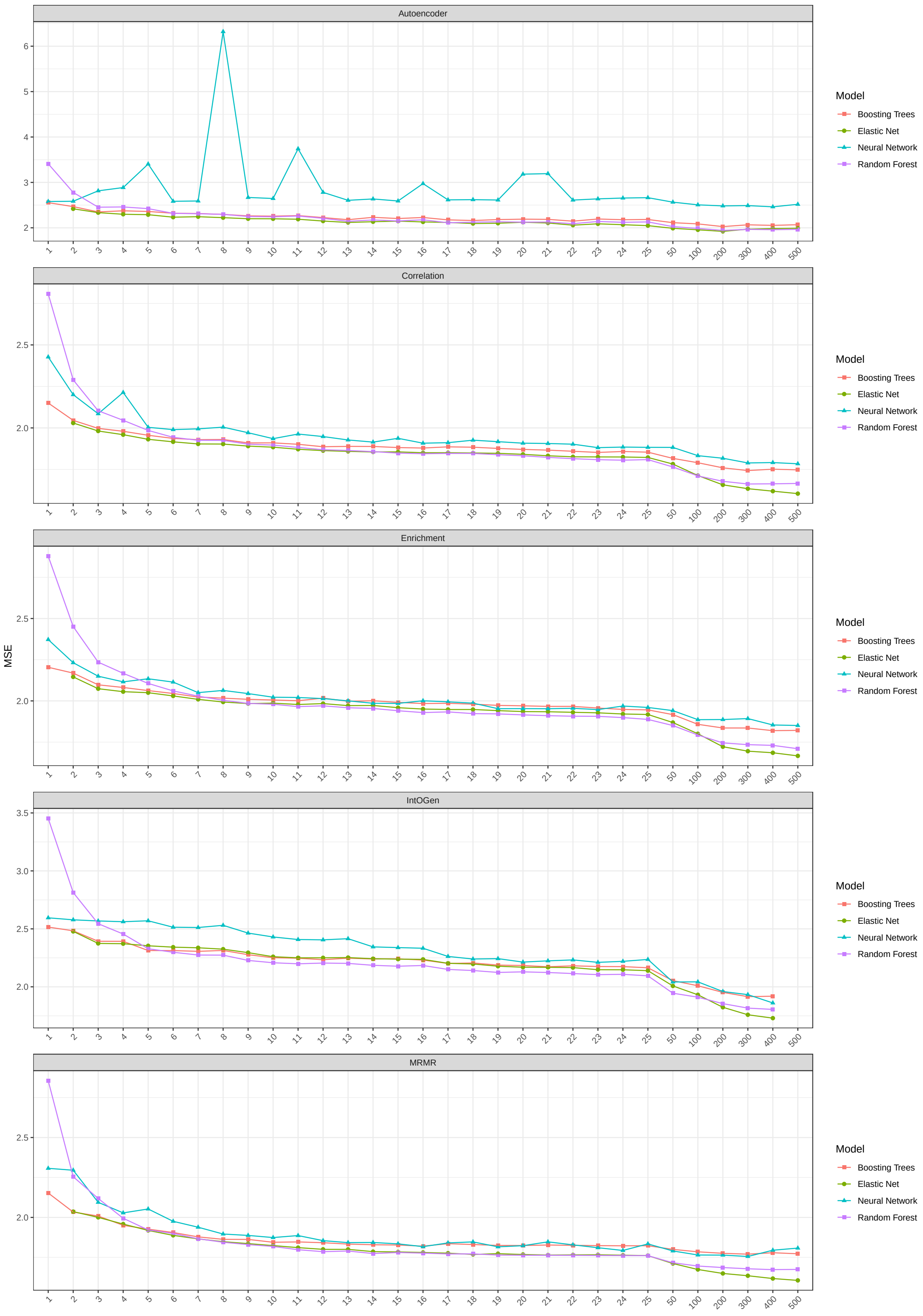

Figure 6: Figure continues on next page.

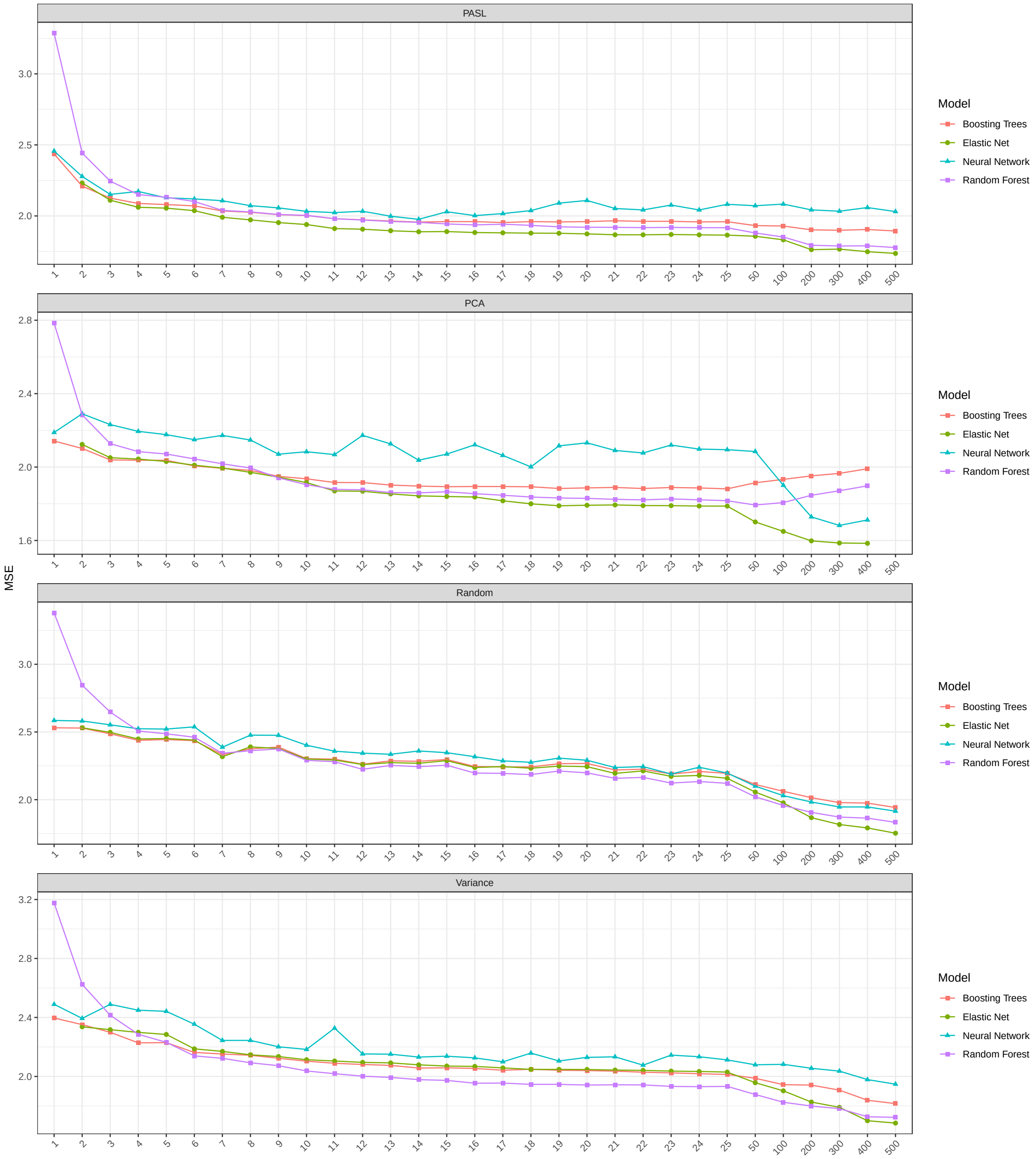

Figure 6: Average test MSEs for each DR algorithm for 50 drugs with most cell lines. This figure depicts the test MSEs averaged over the 50 drugs with most cell lines (c.f. Table 2) for each combination of DR algorithm, ML method and number of input features. Each plot corresponds to one DR algorithm, where the x-axis denotes the number of input features, the y-axis denotes the mean test MSE and the coloring represents the different ML methods.

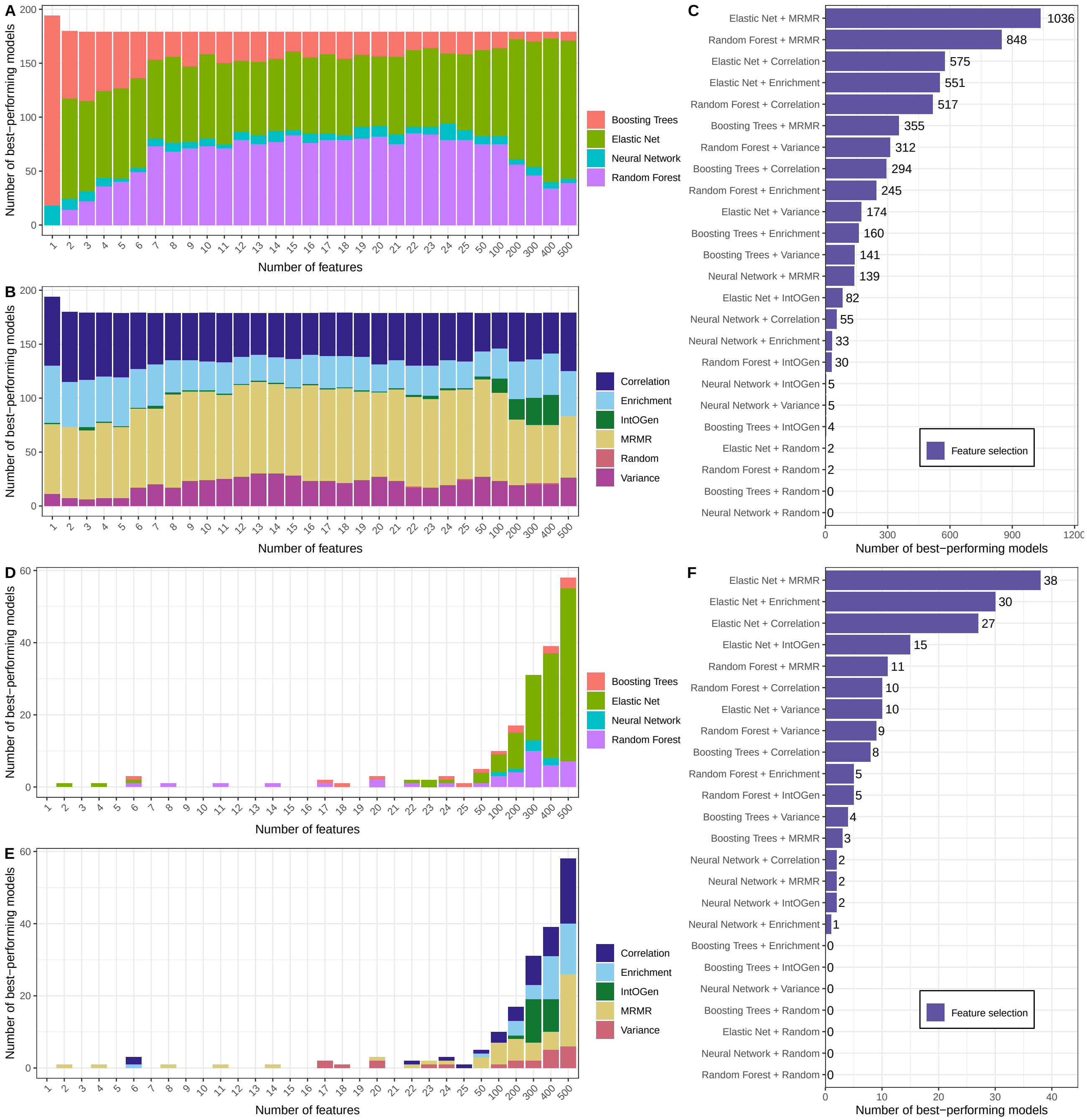

Figure 7: Best-performing models for each drug and number of input features using only FS methods. Sub-figure A and B, respectively, show how often each ML algorithm and FS method yielded the smallest test MSE after performing a hyperparameter tuning (5-fold CV) to determine the best performing model for each combination of drug, ML algorithm, FS method and number of input features. Sub-figure C shows how many times a given combination of ML algorithm and FS method yielded the best results. Subfigures D to F depict the same results, but only the feature number yielding the smallest test MSE for each drug is shown.

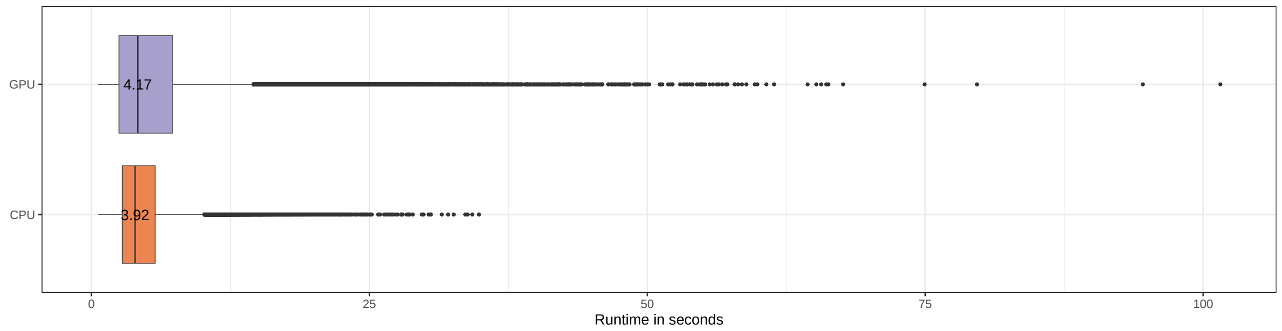

Figure 8: Runtime comparison GPU vs. CPU. This Figure shows the duration of training neural networks (inputs generated using MRMR FS) using either GPU or CPU (c.f. runtime analysis in results section of main manuscript). Runtimes are given in seconds and the median runtime is shown in the purple/orange box (mean runtimes are 5.95 for GPU and 4.65s for CPU).

2 Analyses using a multi-omics and multi-drug deep-learning approach by Chiu et al.

To investigate the impact of different dimension reduction (DR) procedures on a state-of-the-art method for drug sensitivity prediction and to compare the performance of this method to the ML algorithms discussed in the main manuscript, we performed several analyses using a multi-omics multi-drug deep-learning approach by Chiu et al. [17]. In the following, we will briefly present their approach and then describe the details of our analyses, including the used data, models, and DR techniques. Finally, we discuss the analysis results.

2.1 The approach by Chiu et al.

Chiu et al. developed a multi-omics deep neural network (DNN) for drug sensitivity prediction that predicts the IC50 of multiple drugs simultaneously. The inputs consist of gene expression values and binary mutation data for one cell line. Using one expression-autoencoder and one mutation-autoencoder, these inputs are projected into a lower dimension of $k = 64$ features each. The autoencoders were pre-trained using data from tumor samples obtained from *The Cancer Genome Atlas* (TCGA, https://www.cancer.gov/tcga). The pre-trained encoders are then connected to a DNN with drug-specific output nodes. The entire model was trained and evaluated using cell line data from the *Cancer Cell Line Encyclopedia* (CCLE) [34].

2.2 Data processing

To apply the approach by Chiu et al. to the GDSC data and to compare its performance to that of other ML models, we prepared the data as follows:

- *Gene expression data:* We employ the same gene expression data as described in the main manuscript.
- *Mutation data:* We generated a binary mutation matrix $M_{cells \times genes}$, where each entry $M_{c,g}$ denotes whether gene g is mutated in cell line c ($M_{c,g} = 1$) or not ($M_{c,g} = 0$). We obtained coding point mutations of the GDSC cell lines from v99 of the COSMIC cell line project (file: CellLinesProject_GenomeScreensMutant_v99_GRCh37.tsv). In accordance with Chiu et al., we did not consider synonymous mutations.
- *Drug response data:* We employ the same drug response data as described in the main manuscript. However, since the model by Chiu et al. makes predictions for multiple drugs simultaneously, it requires data where each investigated cell line provides IC50 values for each investigated drug. In the GDSC, not all cell lines have been screened against all drugs. To determine a maximal but complete subset of cell lines and drugs for our analyses, we applied an integer linear program (ILP) that we previously described in Supplement 1 of [11]. This ILP determined a set consisting of 600 cell lines and 170 drugs.

- *Splitting into training and test data:* We randomly split the 600 cell lines with available expression, mutation, and drug response data into a training set (80%) and a test set (20%).

2.3 Model architecture and hyperparameters

- *Approach by Chiu et al.:* We used the same model architecture and hyperparameters that Chiu et al. employ in their code (https://github.com/chenlabgccri/DeepDR). However, we did not only investigate a dimension reduction to $k = 64$ features for each omics-type but different feature numbers between 1 and 500 (c.f. Supplementary Figure 9). Additionally, we investigated the performance when either the expression-encoder or the mutation-encoder was omitted from the model. Note that both the CCLE and TCGA data employed by Chiu et al. measure gene expression using RNA-seq, while the GDSC used in our manuscript relies on microarrays. Consequently, pre-training using TCGA data was not possible for our analyses, so we used the training samples for pre-training instead. According to Chiu et al., TCGA pre-trained models resulted in the best performance, but even using randomly initialized encoders outperformed all comparable analyses without pre-training [17]. Consequently, pre-training using the training cell lines should outperform most comparable alternatives including random initialization, when TCGA pre-training is not possible.

- *Elastic net and random forest:* We trained drug-specific elastic net and random forest models using the same training and test cell lines as described in Section 2.2. We tuned the same hyperparameters as described in the main manuscript (see Table 1) using a 5-fold cross-validation on the training data.

2.4 Investigated DR approaches:

In addition to the autoencoders employed by Chiu et al., we investigated two further DR methods:

- *Principal component analysis (PCA):* We performed PCA on the gene expression data as described in the main manuscript.

- *Correlation-based feature selection:* For the gene expression features, we employed the same correlation-based feature selection as described in the main manuscript using Pearson correlation coefficients (PCC). Since the mutation data is not continuous but binary (c.f. Section 2.2), we used Matthew's correlation coefficient (MCC) instead of PCC for this data: For each drug, we selected the k genes with the highest absolute MCC between the mutation profile of each gene and the binarized IC50 values of the corresponding cell lines. IC50 values were binarized using drug-specific thresholds obtained from a procedure described in [2].

- *Multi-drug feature sets:* Since the approach by Chiu et al. is a multi-drug model, we cannot use drug-specific feature sets but need one feature set for all drugs. Since PCA does not make use of any drug response values, it yields the same features for all investigated drugs, which can directly be used by the approach of Chiu et al. However, we slightly adapted the correlation-based methods presented above: We generated feature sets of different sizes by subsequently including the top 1,2,...,f most correlated features for each drug, as long as the size of resulting feature sets did not exceed 500. For gene expression data, we were able to include the top $f = 13$ most correlated features for each drug, resulting in 13 feature sets (feature numbers 71, 116, 151, 191, 226, 261, 296, 323, 357, 396, 422, 454, 487). For mutation data, we were only able to include the top $f = 4$ most correlated features for each drug, resulting in four feature sets (feature numbers 136, 253, 364, 454). Since some features are among the top features for multiple drugs, the size of the resulting sets is not necessarily a multiple of the drug number (170).

2.5 Results

The results of our analyses are shown in Supplementary Figures 9 to 11. Several observations can be made (see main manuscript for further discussion):

- Models based on the approach by Chiu et al. with autoencoders perform worse than all other approaches for most $k \leq 50$. Potentially, these models fail to learn in the number of training epochs chosen by Chiu et

al. (100 epochs for autoencoders, 50 epochs for final model) and, consequently, only predict the mean IC50 over all drugs and training cell lines.

- All models based on the approach by Chiu et al. have noticeable discrepancies in the mean test MSE for different k (i.e., the curve shape in Figure 9 is unstable). A similar phenomenon was also observed for neural networks in our analyses (c.f. Figure 1 in the main manuscript and Figures 2 to 6 in this Supplement). This might again be caused by network training not converging in the set number of training epochs or by the optimization not being able to leave a local minimum.

- Expression features outperform mutation features for all models (i.e., the models by Chiu et al., elastic nets, and random forests).

- Elastic nets and random forests using mutation features seem to be unable to learn since the test MSE does barely vary across different k.

- Using PCA instead of autoencoders strongly improves the performance of models based on the approach by Chiu et al. for small k. Using correlation-based expression features results in the best test MSEs out of all models based on the approach by Chiu et al.

- Elastic net and random forest models using expression-based features significantly outperform all other models.

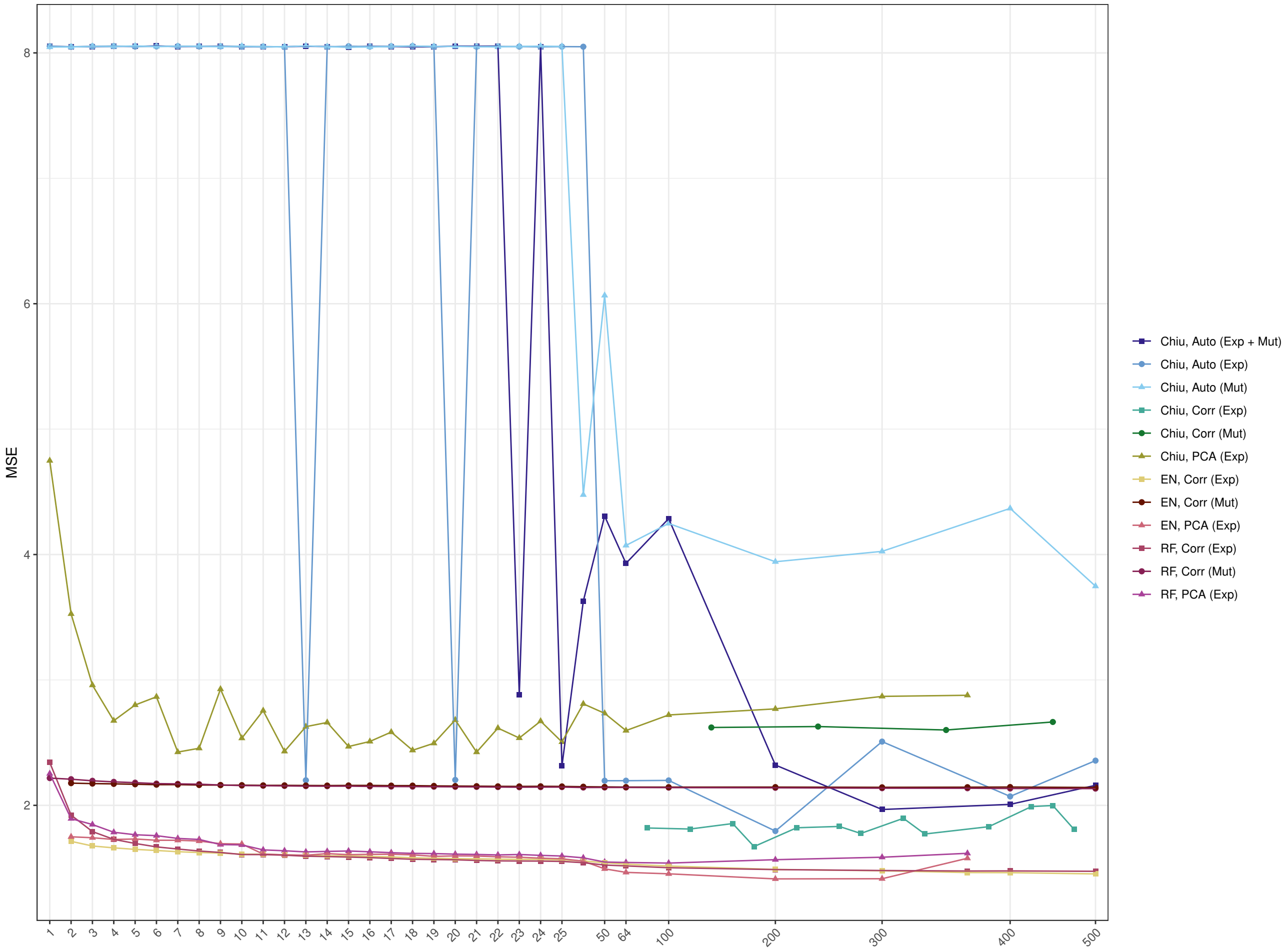

Figure 9: Performance of the approach by Chiu et al. in comparison to other models. This figure depicts the results of applying the prediction approach by Chiu et al. [17] and some variations of it to 170 drugs from the GDSC. Additionally, the performance of single-drug elastic nets and random forests trained on the same data is shown. The x-axis denotes the number of input features of each data type, the y-axis denotes the mean test MSE averaged over all drugs, and the coloring represents the different approaches. Note that for the multi-omics model by Chiu et al. (Chiu, Auto (Exp + Mut)), the number of features is twice as large as denoted by the x-axis, since two omics types are used. The legend lists all approaches using the following abbreviations: EN - elastic net, RF - random forest, Auto - autoencoder, Corr - correlation. In brackets, each model's data types are specified: Exp - gene expression, Mut - mutation.

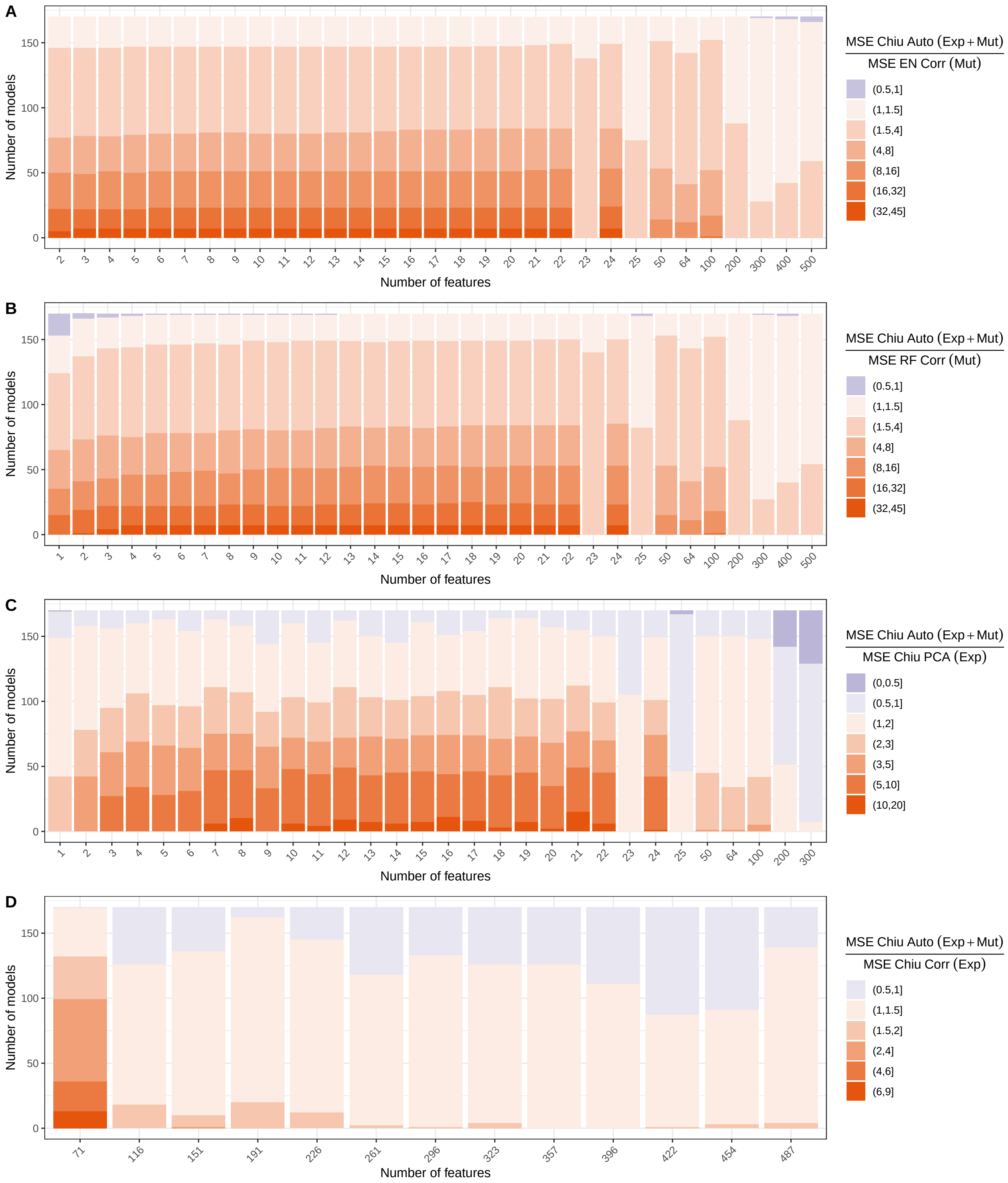

Figure 10: Performance comparison the approach by Chiu et al. to models with different ML/DR methods. Each sub-figure depicts a comparison of test MSEs for two model types, where the MSE for one type is divided by the MSE of the other: Sub-figures A and B compare the test MSE of the approach by Chiu et al. for each drug and k to the MSE of drug-specific elastic nets (EN) and random forests (RF), respectively. Both EN and RF were trained using correlation-based gene expression features. Sub-figures C and D compare the test MSE of the approach by Chiu et al. to adapted versions of their approach using PCA or correlation-based features instead of autoencoders, respectively. Note that for the multi-omics model by Chiu et al. (Chiu Auto (Exp + Mut)), the number of features is twice as large as denoted by the x-axis, since two omics types are used.

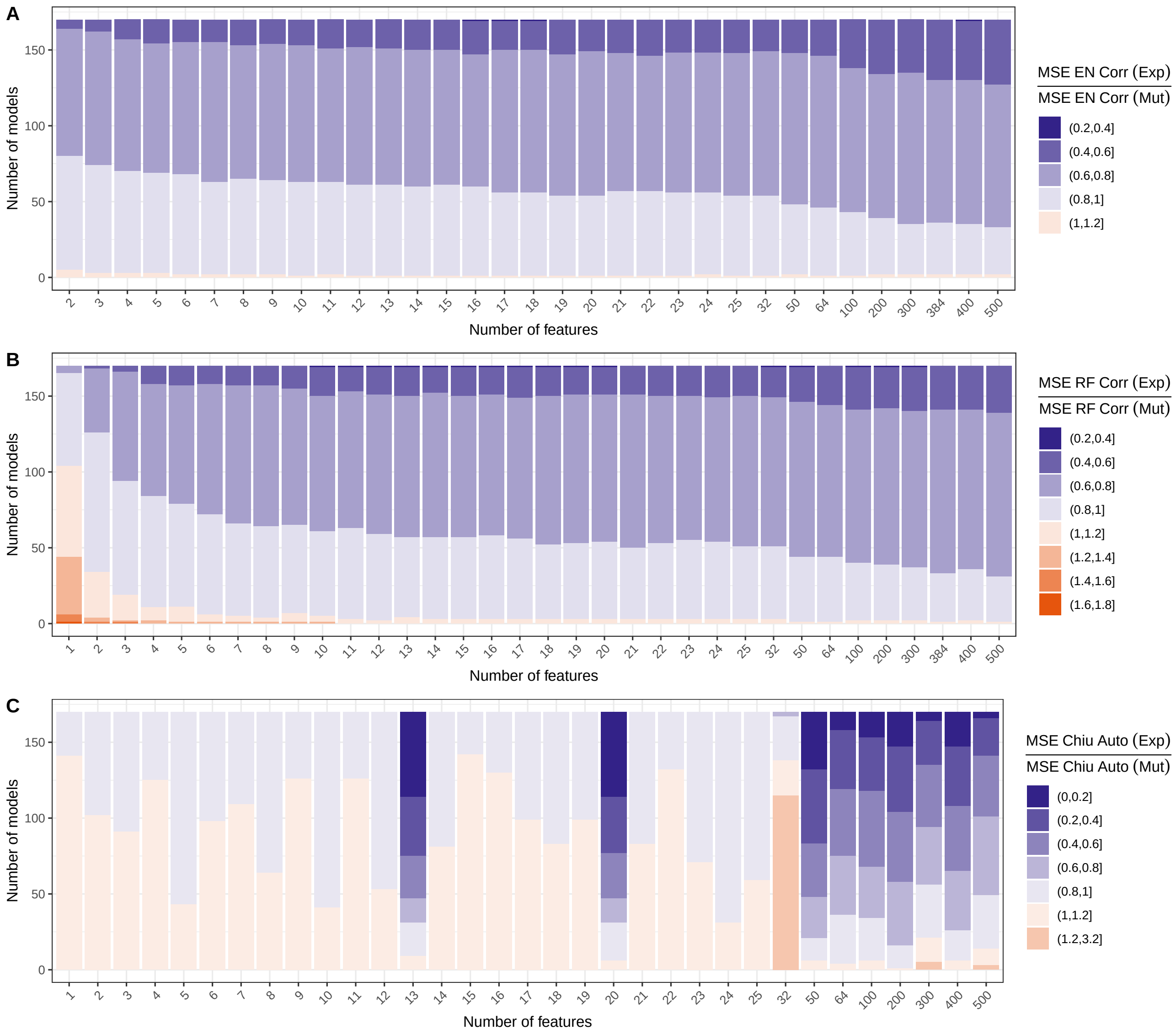

Figure 11: Performance comparison of gene expression and mutation features. Each sub-figure depicts a comparison of test MSEs for two model types, where the MSE for one type is divided by the MSE of the other. One model type uses gene expression features, the other uses mutation features. Sub-figures A shows results for elastic nets, sub-figure B shows results for random forests, and sub-figure C shows results for the approach by Chiu et al. employing either only the expression encoder or only the mutation encoder.

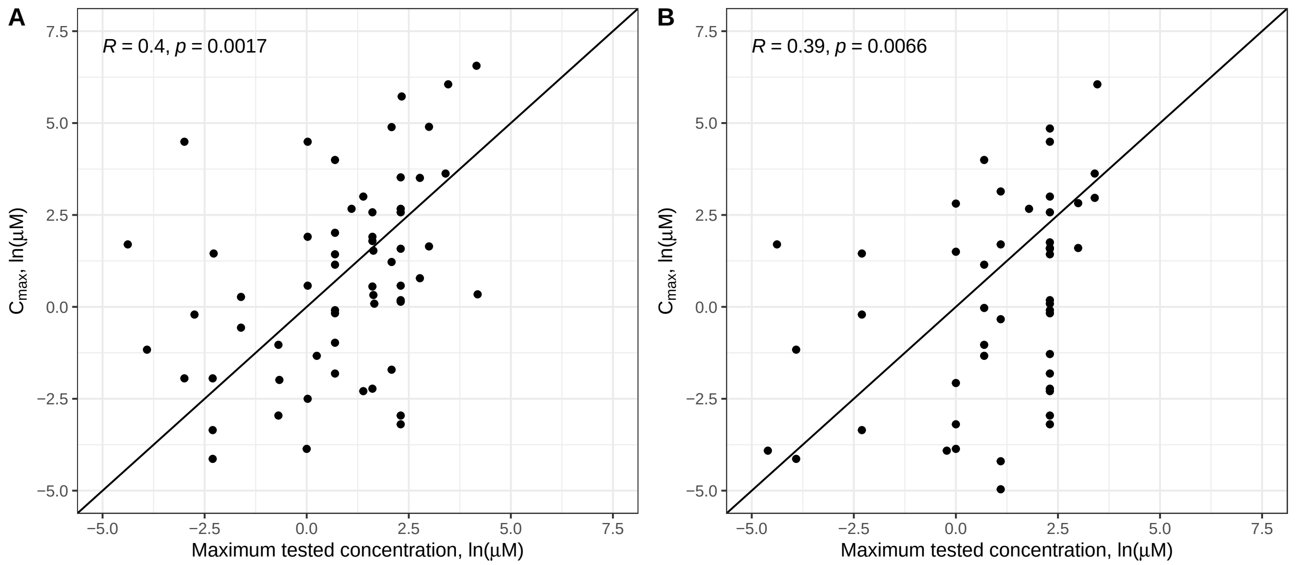

Figure 12: Comparison of C_{max} values and maximum tested drug concentrations. This figure depicts the C_{max} concentrations for 60 drugs from GDSC1 (A) and 47 drugs from GDSC2 (B) in comparison to the maximum screened concentrations (which are used to determine the screened drug concentration ranges by repeated dilution) as provided by the GDSC. The C_{max} concentrations were obtained from [35] and denote the peak plasma concentration of a drug after administering the highest clinically recommended dose.

References

[1] Lei Deng, Yideng Cai, Wenhao Zhang, Wenyi Yang, Bo Gao, and Hui Liu. Pathway-guided deep neural network toward interpretable and predictive modeling of drug sensitivity. *Journal of Chemical Information and Modeling*, 60(10):4497–4505, 2020.

[2] T. A. Knijnenburg, G. W. Klau, F. Iorio, M. J. Garnett, U. McDermott, I. Shmulevich, and L. F. A. Wessels. Logic models to predict continuous outputs based on binary inputs with an application to personalized cancer therapy. *Scientific Reports*, 6(1):1–14, 2016.

[3] M. P. Menden, F. Iorio, M. Garnett, U. McDermott, C. H. Benes, P. J. Ballester, and J. Saez-Rodriguez. Machine learning prediction of cancer cell sensitivity to drugs based on genomic and chemical properties. *PLoS ONE*, 8(4):e61318, 2013.

[4] Kerstin Lenhof, Nico Gerstner, Tim Kehl, Lea Eckhart, Lara Schneider, and Hans-Peter Lenhof. Merida: a novel boolean logic-based integer linear program for personalized cancer therapy. *Bioinformatics*, 37(21):3881–3888, 2021.

[5] Yoosup Chang, Hyejin Park, Hyun-Jin Yang, Seungju Lee, Kwee-Yum Lee, Tae Soon Kim, Jongsun Jung, and Jae-Min Shin. Cancer drug response profile scan (cdrscan): a deep learning model that predicts drug effectiveness from cancer genomic signature. *Scientific reports*, 8(1):8857, 2018.

[6] Mingyang Liu, Xiaotong Shen, and Wei Pan. Deep reinforcement learning for personalized treatment recommendation. *Statistics in medicine*, 41(20):4034–4056, 2022.

[7] Zachary Stanfield, Mustafa Coşkun, and Mehmet Koyutürk. Drug response prediction as a link prediction problem. *Scientific reports*, 7(1):40321, 2017.

[8] Ladislav Rampášek, Daniel Hidru, Petr Smirnov, Benjamin Haibe-Kains, and Anna Goldenberg. Dr.VAE: improving drug response prediction via modeling of drug perturbation effects. *Bioinformatics*, 35(19):3743–3751, 03 2019.

[9] Yun Fang, Peirong Xu, Jialiang Yang, and Yufang Qin. A quantile regression forest based method to predict drug response and assess prediction reliability. *PLoS One*, 13(10):e0205155, 2018.

[10] Kerstin Lenhof, Lea Eckhart, Nico Gerstner, Tim Kehl, and Hans-Peter Lenhof. Simultaneous regression and classification for drug sensitivity prediction using an advanced random forest method. *Scientific Reports*, 12(1):13458, 2022.

[11] Kerstin Lenhof, Lea Eckhart, Lisa-Marie Rolli, Andrea Volkamer, and Hans-Peter Lenhof. Reliable anti-cancer drug sensitivity prediction and prioritization. *Research Square preprint*, 2023.

[12] K. Matlock, C. De Niz, R. Rahman, S. Ghosh, and R. Pal. Investigation of model stacking for drug sensitivity prediction. *BMC Bioinformatics*, 19(3):21–33, 2018.

[13] R. Rahman, K. Matlock, S. Ghosh, and R. Pal. Heterogeneity aware random forest for drug sensitivity prediction. *Scientific Reports*, 7(1):1–11, 2017.

[14] Ran Su, Xinyi Liu, Leyi Wei, and Quan Zou. Deep-resp-forest: a deep forest model to predict anti-cancer drug response. *Methods*, 166:91–102, 2019.

[15] Amrita Basu, Ritwik Mitra, Han Liu, Stuart L Schreiber, and Paul A Clemons. Rwen: response-weighted elastic net for prediction of chemosensitivity of cancer cell lines. *Bioinformatics*, 34(19):3332–3339, 2018.

[16] Ali Oskooei, Matteo Manica, Roland Mathis, and María Rodríguez Martínez. Network-based biased tree ensembles (netbite) for drug sensitivity prediction and drug sensitivity biomarker identification in cancer. *Scientific reports*, 9(1):15918, 2019.

[17] Y.-C. Chiu, H.-I. H. Chen, T. Zhang, S. Zhang, A. Gorthi, L.-J. Wang, Y. Huang, and Y. Chen. Predicting drug response of tumors from integrated genomic profiles by deep neural networks. *BMC Medical Genomics*, 12(1):143–155, 2019.

[18] L. Wang, X. Li, L. Zhang, and Q. Gao. Improved anticancer drug response prediction in cell lines using matrix factorization with similarity regularization. *BMC Cancer*, 17(1):1–12, 2017.

[19] Y.-C. Tang and A. Gottlieb. Explainable drug sensitivity prediction through cancer pathway enrichment. *Scientific reports*, 11(1):1–10, 2021.

[20] N. Zhang, H. Wang, Y. Fang, J. Wang, X. Zheng, and X. S. Liu. Predicting anticancer drug responses using a dual-layer integrated cell line-drug network model. *PLOS Computational Biology*, 11(9):e1004498, 2015.

[21] Hui Liu, Yan Zhao, Lin Zhang, and Xing Chen. Anti-cancer drug response prediction using neighbor-based collaborative filtering with global effect removal. *Molecular Therapy - Nucleic Acids*, 13:303–311, 2018.

[22] Fei Zhang, Minghui Wang, Jianing Xi, Jianghong Yang, and Ao Li. A novel heterogeneous network-based method for drug response prediction in cancer cell lines. *Scientific reports*, 8(1):3355, 2018.

[23] Fatemeh Ahmadi Moughari and Changiz Eslahchi. Adrml: anticancer drug response prediction using manifold learning. *Scientific reports*, 10(1):14245, 2020.

[24] Xiao He, Lukas Folkman, and Karsten Borgwardt. Kernelized rank learning for personalized drug recommendation. *Bioinformatics*, 34(16):2808–2816, 03 2018.

[25] R. Rahman and R. Pal. Analyzing drug sensitivity prediction based on dose response curve characteristics. In *IEEE-EMBS International Conference on Biomedical and Health Informatics (BHI)*, pages 140–143, 2016.

[26] Tuan Nguyen, Giang T. T. Nguyen, Thin Nguyen, and Duc-Hau Le. Graph convolutional networks for drug response prediction. *IEEE/ACM Transactions on Computational Biology and Bioinformatics*, 19(1):146–154, 2022.

[27] Xuan Liu, Congzhi Song, Feng Huang, Haitao Fu, Wenjie Xiao, and Wen Zhang. Graphcdr: a graph neural network method with contrastive learning for cancer drug response prediction. *Briefings in Bioinformatics*, 23(1):bbab457, 2022.

[28] Kanggeun Lee, Dongbin Cho, Jinho Jang, Kang Choi, Hyoung-oh Jeong, Jiwon Seo, Won-Ki Jeong, and Semin Lee. Ramp: response-aware multi-task learning with contrastive regularization for cancer drug response prediction. *Briefings in Bioinformatics*, 24(1):bbac504, 2023.

[29] Hossein Sharifi-Noghabi, Olga Zolotareva, Colin C Collins, and Martin Ester. Moli: multi-omics late integration with deep neural networks for drug response prediction. *Bioinformatics*, 35(14):i501–i509, 2019.

[30] Xiaoxiao Cheng, Chong Dai, Yuqi Wen, Xiaoqi Wang, Xiaochen Bo, Song He, and Shaoliang Peng. Nerd: a multichannel neural network to predict cellular response of drugs by integrating multidimensional data. *BMC medicine*, 20(1):368, 2022.

[31] Hong Wang, Chong Dai, Yuqi Wen, Xiaoqi Wang, Wenjuan Liu, Song He, Xiaochen Bo, and Shaoliang Peng. Gadrp: graph convolutional networks and autoencoders for cancer drug response prediction. *Briefings in Bioinformatics*, 24(1):bbac501, 2023.

[32] Peilin Jia, Ruifeng Hu, and Zhongming Zhao. Benchmark of embedding-based methods for accurate and transferable prediction of drug response. *Briefings in Bioinformatics*, 24(3):bbad098, 2023.

[33] N Kwak and C.-H. Choi. Input feature selection for classification problems. *IEEE transactions on neural networks*, 13(1):143 – 159, 2002.

[34] M. Ghandi, F. W. Huang, J. Jané-Valbuena, G. V. Kryukov, C. C. Lo, E. R. McDonald, J. Barretina, E. T. Gelfand, C. M. Bielski, and H. et al. Li. Next-generation characterization of the cancer cell line encyclopedia. *Nature*, 569(7757):503–508, 2019.

[35] Dane R Liston and Myrtle Davis. Clinically relevant concentrations of anticancer drugs: A guide for nonclinical studiesguide to clinical exposures of anticancer drugs. *Clinical cancer research*, 23(14):3489–3498, 2017.
